# Eclipsed and Twisted Excimers of Pyrene and 2-Azapyrene: How Nitrogen Substitution Impacts Excimer Emission

**DOI:** 10.3390/molecules29020507

**Published:** 2024-01-19

**Authors:** Yasi Dai, Filippo Rambaldi, Fabrizia Negri

**Affiliations:** 1 Department of Chemistry “Giacomo Ciamician”, University of Bologna, 40126 Bologna, Italy; yasi.dai2@unibo.it (Y.D.); filippo.rambaldi@studio.unibo.it (F.R.); 2 Center for Chemical Catalysis—C3, Alma Mater Studiorum—Università di Bologna, Via Selmi 2, 40126 Bologna, Italy; 3 Consorzio Interuniversitario Nazionale per la Scienza e Tecnologia dei Materiali (INSTM), Research Unit of Bologna, 40126 Bologna, Italy

**Keywords:** pyrene, 2-azapyrene, excimer, exciton states, polycyclic aromatic hydrocarbons, TD-DFT, diabatization, Frenkel excitons, charge transfer states

## Abstract

Due to their unique photophysical and electronic properties, pyrene and its analogues have been the subject of extensive research in recent decades. The propensity of pyrene and its derivatives to form excimers has found wide application in various fields. Nitrogen-substituted pyrene derivatives display similar photophysical properties, but for them, excimer emission has not been reported to date. Here, we use time-dependent density functional theory (TD-DFT) calculations to investigate the low-lying exciton states of dimers of pyrene and 2-azapyrene. The excimer equilibrium structures are determined and the contribution of charge transfer (*CT*) excitations and intermolecular interactions to the exciton states is disclosed using a diabatization procedure. The study reveals that the dimers formed by the two molecules have quite similar exciton-state patterns, in which the relevant *CT* contributions govern the formation of excimer states, along with the *L_a_*/*L_b_* state inversion. In contrast with pyrene, the dipole–dipole interactions in 2-azapyrene stabilize the dark eclipsed excimer structure and increase the barrier for conversion into a bright twisted excimer. It is suggested that these differences in the nitrogen-substituted derivative might influence the excimer emission properties.

## 1. Introduction

Polycyclic aromatic hydrocarbons (PAHs) are recognized as a highly important class of organic compounds. They have long been, and still are, of interest in many research fields ranging from environmental science, where they are investigated for their role as pollutants [1], to Earth science and astronomy, in which they are of great interest to early Earth and Mars origin-of-life studies [2], and then to organic electronics, where they are used as semiconductors [3,4,5,6,7,8,9]. Among this wide family of chemical species, pyrene emerges as the preferred chromophore in both fundamental and applied photochemical research thanks to its properties, which have found application in many scientific areas [9]. In particular, pyrene and its analogues have been intensively studied in recent decades because of their unique photophysical and electronical properties. The solvent polarity dependence of the fluorescence emission of pyrene has been used extensively for probing local polarity and microenvironmental changes [10]. Specifically, what makes pyrene a fluorescent probe is the high sensitivity of its monomer fluorescence to solvent polarity, resulting in relative intensity changes in selected vibronic bands [10,11]. The propensity of pyrene and its derivatives to readily generate excimers [12], characterized by distinct fluorescence bands for both the monomer and excimer species, has found extensive application in supramolecular design, in exploring the structural properties of proteins, peptides and DNA [13,14] and as sensitizers in photodynamic therapy [15]. For example, pyrene is covalently attached to a residue, and its fluorescence is exploited to investigate the molecular organization or conformation of proteins since the extent of excimer emission can be correlated with the distance between two pyrenes bound to different locations [14]. Beyond its role as a fluorescent probe in sensing applications, pyrene has also demonstrated its utility as an organic semiconductor, finding applications in the fields of materials science and organic electronics [9,16,17,18].

As the quest for appealing and adaptable organic materials continues, the design of π-conjugated polycyclic (hetero)aromatic hydrocarbons has emerged as a prominent subject of interest in the field of materials science. Since the 16π-electron conjugated polycyclic hydrocarbon exhibits desirable electronic and photophysical properties, doping the pyrene core with more electronegative atoms, like nitrogen, has become a novel efficient strategy for fine-tuning such properties [19,20,21,22,23,24]. The introduction of a nitrogen atom in place of a carbon within a peri-fused pyrene framework brings about a substantial modification and enhancement of the properties, leading to novel and diverse applications as materials in organic photovoltaics (OPVs), organic light-emitting diodes (OLEDs) and organic field-effect transistors (OFETs) [25]. Among several nitrogen-substituted pyrenes, the photophysical properties of monosubstituted derivatives have been carefully investigated [20,21,24,26,27]. The absorption spectrum of 2-azapyrene in ethanol resembles that of pyrene [27]. The emission spectra of mono-substituted azapyrenes were measured at low concentrations, in solvents of different polarity to assess their application as fluorescent probes. However, while the main vibronic bands reveal remarkable similarities to pyrene monomer fluorescence [26,27], no solvent enhancement was found. Absorption and emission spectra similar to those of pyrene have been measured in dichloromethane, at a low concentration, also for substituted 2-azapyrene derivatives [20]. Thus, in contrast with pyrene, the excimer emission from azapyrenes has not been reported so far, probably because of the low concentrations employed in the experimental studies.

**Figure 1:**
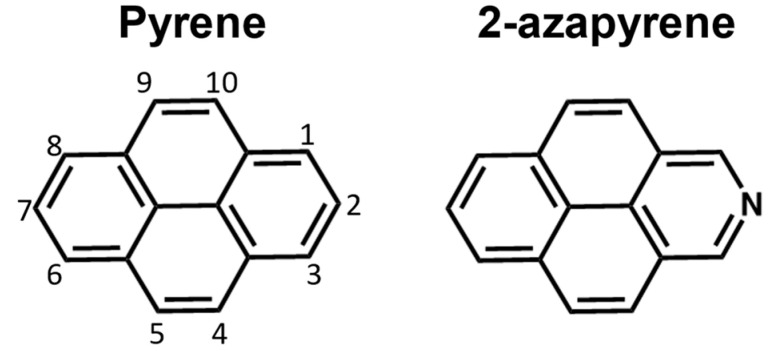
Pyrene and 2-azapyrene considered in this work.

Computational investigations are fundamental to unveiling the mechanisms that govern the photoinduced processes of π-conjugated chromophores and exploiting their properties in functional photonic materials. Several computational investigations have focused on the determination of the equilibrium structure and stabilization of pyrene’s excimer [28,29,30,31,32,33,34]. Concerning nitrogen-substituted derivatives, a systematic computational analysis on the effect of the position and nature (graphitic vs. pyridinic) in di-azapyrene has been reported [35]. Remarkably, there has been comparatively less computational attention on the single N-substitution of pyrene and its excimers. In this work, we seek to fill this gap by assessing the similarities and differences between pyrene and 2-azapyrene (Figure 1), a prototypical N-substituted pyrene, by investigating their ground- and excited-state dimeric structures, paying attention to the formation of excimer states. The investigation of dimers is addressed using two approaches, both based on quantum chemical investigations: first, direct ground- and exciton-state geometry optimization is carried out to determine the equilibrium structures and the stability of the excimer structures. Excimer formation is explored along the intermolecular long-axis translation coordinate and along the intermolecular rotational coordinate. As the exciton states of molecular aggregates are linear combinations of local (intramolecular) excitations ( LEs ) and charge transfer ( CT ) (i.e., intermolecular) excitations, such intramolecular and intermolecular excitations represent a convenient set of diabatic states that can be exploited to analyze the character of the exciton/excimer states [36,37,38,39,40,41,42,43,44,45,46,47,48,49,50]. Thus, seeking to uncover the nature of the excimer states, we use a diabatization procedure to capture the contribution of the CT states, crucial in the formation of excimers [51,52,53], and to unveil the most relevant intermolecular interactions.

## 2. Results and Discussion

### 2.1. Orbital Nature of Low-Lying Excited States of Pyrene and 2-Azapyrene

**Figure 2:**
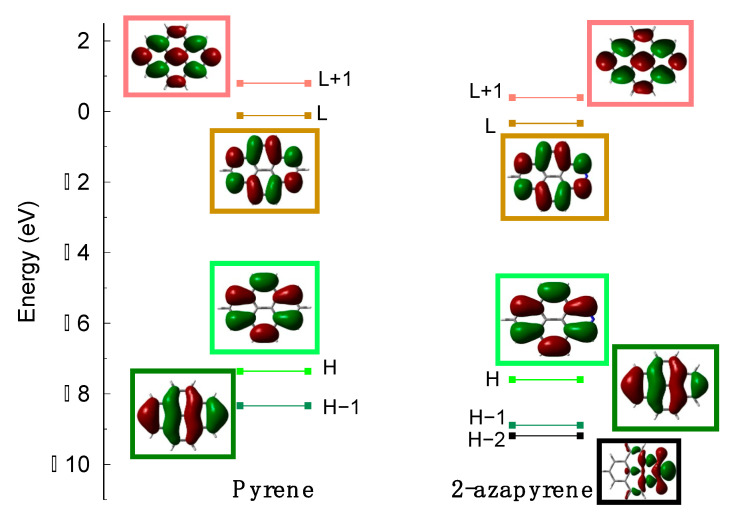
Frontier molecular orbital shapes and energies of (**left**) pyrene and (**right**) 2-azapyrene, computed at ωB97X-D/def2-SVP level (for simplicity, HOMO and LUMO are abbreviated as H and L).

Pyrene and 2-azapyrene differ only according to nitrogen substitution. Notably, the substitution of a CH group with a nitrogen atom, resulting in a pyridinic configuration, implies that the lone-pair orbital of the nitrogen is located in the molecular plane, and the number of electrons of the π-system is identical to the unsubstituted system. However, the presence of the heteroatom reduces the symmetry from D_2h_ to C_2v_ and, due to the higher electronegativity of nitrogen compared to carbon, 2-azapyrene acquires a dipole moment, which is computed to be 2.7 Debye at the ground-state optimized geometry (ωB97X-D/def2-SVP level). The frontier molecular orbitals (MOs) of 2-azapyrene are almost identical to those of pyrene (Figure 2) and the nitrogen lone-pair orbital is found below the HOMO−1.

As found for other PAHs, the lowest lying excited states of pyrene and 2-azapyrene (Table S1) are dominated by excitations encompassing at least two occupied (HOMO and HOMO−1) and two unoccupied (LUMO and LUMO + 1) MOs. These excitations determine four low-lying excited states: $L_{a},\;L_{b},\;B_{a}{,B}_{b}$ in Platt’s notation [54]. As demonstrated previously for naphthalene [55], the ωB97X-D functional (Section 3) slightly overestimates the excitation energies but correctly predicts, also for pyrene, that the lowest excited state is the $L_{b}$ state, namely the state described as a linear combination of the HOMO−1→LUMO and HOMO→LUMO + 1 excitations, followed at higher energy by the $L_{a}$ state, described by the HOMO→LUMO excitation, in agreement with the experimental data [56,57]. The experimental absorption spectrum of 2-azapyrene is very similar to that of pyrene [27] with the lowest-energy band followed by a more intense transition, which can be assigned to the $L_{b}$ and $L_{a}$ states, respectively. The $L_{b}$ nature of the lowest-energy state is supported also by a more recent experimental study on phenyl-substituted 2-azapyrene [20]. In agreement with the experimental data, we computed the same order of states as for pyrene (Table S1). The identification of the nature ( $L_{a}$ or $L_{b}$ ) of the lowest lying excited states of the isolated monomers is important to characterize the lowest exciton states and excimers of the molecular dimers discussed in the following sections.

### 2.2. Ground-State Dimer Structures

**Figure 3:**
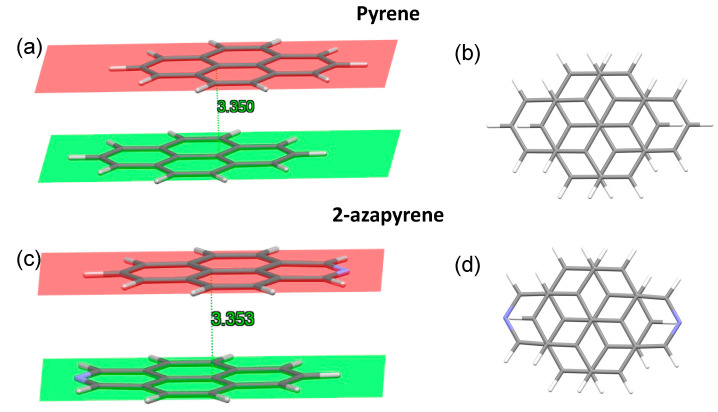
Side and front view of the optimized ground-state structures, and interplanar distances in pyrene (**a**,**b**) and 2-azapyrene (**c**,**d**) dimers, from ωB97X-D/def2-SVP calculations. The molecular planes are determined using Mercury [58] over the full set of atoms forming each monomer.

Calculations predict a minimum energy structure for the ground-state dimer of pyrene and 2-azapyrene in which the two monomers are displaced along the longitudinal translation coordinate, in agreement with previous calculations on pyrene reported in the literature [29,30,31,33]. Interestingly, both optimized structures belong to the C_2h_ symmetry point group and are parallel-displaced along the longitudinal translation coordinate, with a similar interplanar distance, evaluated using Mercury [58], of ca. 3.35 Å (Figure 3). In the case of pyrene, this value is in good agreement with previous calculations (3.45 Å [31], 3.32 Å [30], 3.38 Å [29], 3.52 Å [33]) and with the X-ray structure of pyrene crystals (3.53 Å [59]). 

Notably, the parallel shift in the two pyrene units with respect to each other has been attributed to the minimization of the electrostatic repulsions between opposite C-C bonds [60]. In addition, recent investigations have proposed a molecular orbital-based model to rationalize the ground-state aggregate structures in terms of the exchange–repulsion contribution to the total interaction energy [61]. Thus, the similar parallel shift displaced ground-state structure of the dimers of pyrene and 2-azapyrene is not unexpected given their comparable orbital nature. As a minor difference with pyrene, we note that the nitrogen doping is responsible for a more marked bending of the two molecules of 2-azapyrene. This can be appreciated by considering the interplanar distances, which, for the two planes, are determined by considering only the six central carbon atoms (Figures S1 and S2). Such interplanar distances are larger than those reported in Figure 3 due to the bending: compare 3.386 Å for 2-azapyrene and 3.374 Å for pyrene. The larger distance of 2-azapyrene is compensated for by the smaller distances for the atoms at the end of the long molecular axis: compare 3.315 Å for 2-azapyrene in Figure S2 with 3.334 Å for pyrene in Figure S1. These values show that the terminal part of each 2-azapyrene is more bent toward the other molecule, compared to the pyrene dimer, possibly to maximize the dipole–dipole interaction.

The effect of the dipole moment in 2-azapyrene emerges also from the evaluation of the complexation energy of the ground-state dimer, computed at the optimized geometry, with or without including basis set superposition error (BSSE) [62]. The BSSE-corrected complexation energy of pyrene is computed to be −14.2 kcal/mol compared to the larger (in absolute value) −15.1 kcal/mol for 2-azapyrene. A similar difference is found without BSSE correction: −17.3 kcal/mol and −18.3 kcal/mol for pyrene and 2-azapyrene, respectively. To better assess the nature of these stabilizations, we have examined the empirical dispersion contribution to the complexation energy of the dimers. These are very similar and amount to −17.2 kcal/mol/−17.1 kcal/mol for pyrene/2-azapyrene, respectively. While for pyrene the dispersion energy contribution accounts almost completely for the (non-BSSE-corrected) complexation energy, for 2-azapyrene, the computed (non-BSSE-corrected) complexation is larger (in absolute value) by more than a kcal/mol. The exceeding stabilization in the case of 2-azapyrene is consistent with the additional contribution of electrostatic interactions. A rough estimate of the electrostatic contribution, via simple dipole–dipole interaction (using the computed dipole moment and a distance of 3.35 Å between the two monomers), results indeed in about −2.8 kcal/mol. Finally, we note that the large (in absolute values) computed complexation energies do not include the unfavorable entropic contribution associated with the formation of the dimer. To estimate the role of entropic effects, we also determined the free energy associated with complexation at 298 K, by subtracting the computed free energy of the monomers from that of the dimer and found −5.0 kcal/mol and −5.4 kcal/mol for pyrene and 2-azapyrene, respectively. These values are not BSSE-corrected since counterpoise calculations of free energy stabilization cannot be conducted using Gaussian16. Nevertheless, assuming a BSSE correction of ca. 3 kcal/mol, as computed for the internal energy, this implies an effective stability of the ground-state dimer of about 2 kcal/mol. 

### 2.3. Eclipsed Excimer Structures 

**Figure 4:**
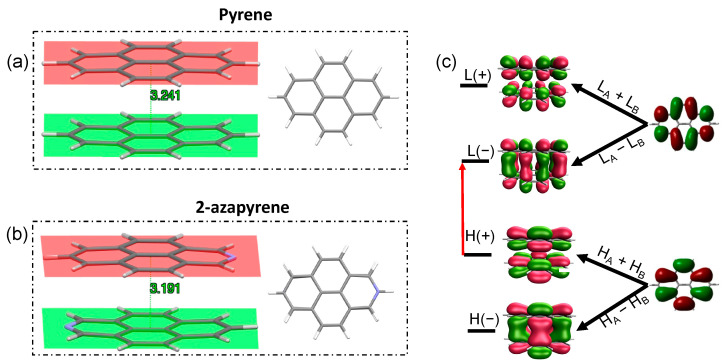
(**a**,**b**) Side and front view of the optimized eclipsed excimer-state structures, along with interplanar distances, in pyrene (**a**) and 2-azapyrene (**b**) dimers (from TD-ωB97X-D/def2-SVP calculations). (**c**) The labels (H(+/−) and L(+/−)) and the shapes of the frontier orbitals delocalized on the dimer indicating their linear combinations of monomer H_A,B_ and L_A,B_ orbitals. The red arrow represents the H(+)→L(−) excitation dominating the excimer wavefunction. (HOMO and LUMO abbreviated as H and L).

The geometry optimization of the excimer state, starting from the ground-state dimer structure, leads to an eclipsed structure not only for pyrene, which is in agreement with previous investigations on pyrene excimers [29,31], but also for 2-azapyrene. The eclipsed geometry and the remarkably reduced interplanar distance, compared to the ground-state structure (Figure 4), is easily rationalized by the wavefunction of the optimized excimers, both dominated by the HOMO(+)-to-LUMO(−) excitation, where (+) or (−) refer to the linear combinations of the monomer’s HOMOs or LUMOs. The strongly bonding nature of the LUMO(−) [29,31,63] favors the eclipsed geometry and its reduced interplanar distance. Furthermore, because the monomer orbitals involved are the HOMO and the LUMO, this implies that the excimer originates from the $L_{a}$ state and not from the lowest-energy excited state of the monomer, which is of $L_{b}$ type for both pyrene and 2-azapyrene. Thus, similarly to naphthalene, an inversion of the two lowest excited states occurs when the aggregate is formed [55,64,65].

The interplanar distance of the eclipsed pyrene excimer, 3.24 Å, is in good agreement with the 3.19 Å distance computed using reference high-level calculations (SCS-CC2/CBS(3,4)) [34]. Our TD-ωB97X-D/def2-SVP result is indeed closer to the reference computed distance than other TD-DFT calculations [31,32,34]: compare, for instance, 3.30 Å for DFT/CAM-B3LYP/6-31G* + D3 [31] or 3.45 Å for TD-PBE0/aug-cc-pVDZ [28]. Compared to pyrene, the eclipsed excimer of 2-azapyrene displays a remarkably shorter interplanar distance of 3.19 Å. In both excimer structures, the molecular units are more remarkably bent out of plane compared to the ground-state structure. Thus, the 3.24 Å and 3.19 Å distances in Figure 4 must be considered average distances with values ranging from 3.22 Å (3.14 Å) to 3.29 Å (3.26 Å) for atoms in the central or in the terminal part of pyrene (2-azapyrene), as can be seen in Figures S3 and S4. As a consequence of the increased dipole–dipole interaction, the excimer geometry of the 2-azapyrene dimer has its constituting units even more bent toward each other with respect to its ground state and closer to each other in comparison to the eclipsed pyrene excimer.

**Figure 5:**
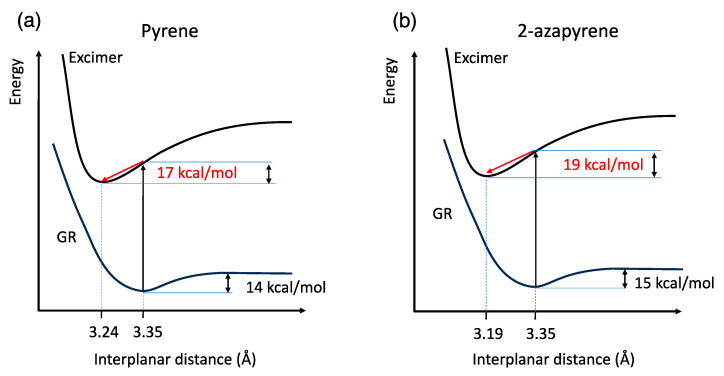
Schematic sketch of (**a**) pyrene and (**b**) 2-azapyrene dimer potential energy curves of the ground and excimer state along the interplanar distance coordinate. From ωB97X-D/def2-SVP and TD-ωB97X-D/def2-SVP calculations. The red arrows represent the stabilization of the excimer from the Franck Condon vertical excitation at the ground-state geometry.

Overall, these calculations can be summarized using the schematic potential energy curves shown in Figure 5, as a function of the interplanar distance. Notably, another consequence of the dipole–dipole interaction in 2-azapyrene is the stabilization of the excimer with respect to the Franck Condon structure (the geometry of ground-state dimer), which is computed to be 2 kcal/mol larger for the nitrogen-substituted PAH compared to pyrene.

### 2.4. Exciton States and Excimer Formation: Evidence from Diabatization

**Figure 6:**
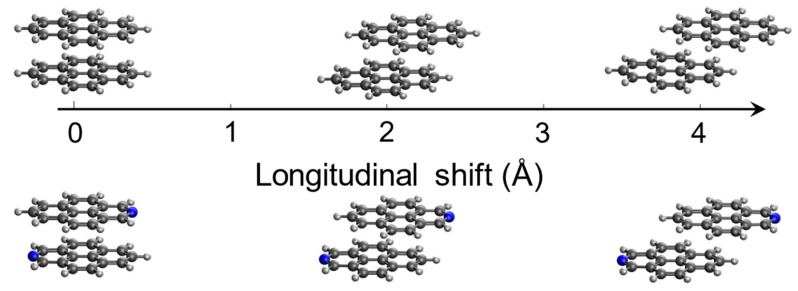
The rigidly displaced pyrene and 2-azapyrene dimers considered in the diabatization procedure. Singlet exciton states were determined starting from the eclipsed configuration and displacing along the interchromophore longitudinal translation (z-axis).

The above discussed geometry optimizations show that the displacement along the longitudinal translation coordinate (Figure 6) effectively leads to the formation of the excimer. Having identified these excimer structures for both pyrene and 2-azapyrene, we seek to analyze, using a diabatization protocol, the nature of these exciton states and the relevant interactions that determine the adiabatic energy profiles along such intermolecular displacement. To this end, a diabatization procedure can be used to determine the linear combination of the LE and CT (diabatic) states corresponding to each computed (adiabatic) exciton state [39,40,46,47,48,49,50,66]. Here, we adopt a diabatization approach that we have developed and applied to unravel the nature of the exciton states of several PAH dimers computed at the TD-DFT level [55,66,67,68]. The details were described in these previous works and can be found in Section 3 and in the Supporting Information section. 

The aggregates investigated here are characterized by a symmetric arrangement of chromophores. In such case, it is more convenient to adopt a set of symmetry-adapted (SA) diabatic states, namely combinations of (neutral) LE states to form Frenkel excitons ( FE ) states and, similarly, combinations of CT states to form charge resonance ( CR ) states [36,69,70] of the appropriate symmetry. The two dimers, for the displacement coordinate discussed here, belong to the symmetry point group $C_{2h}$ . As a result, the most relevant $\pi \pi^{*}\;$ exciton states, along with the FE and CR diabatic states, all belong to $A_{g},A_{u},\;B_{g}$ and $B_{u}$ symmetry representations. It is worth noting that the exciton states derived from the $L_{b}$ state of the monomers belong to $B_{g}$ and $A_{u}$ symmetry, while the exciton states deriving from the $L_{a}$ state of the monomers belong to $A_{g}$ or $B_{u}$ symmetry. 

**Figure 7:**
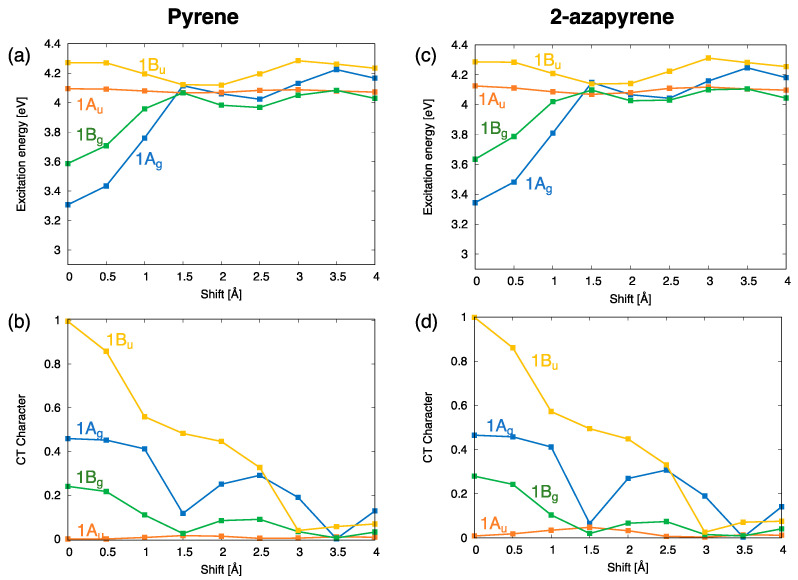
(**a**,**b**) Adiabatic excitation energy profiles and CT character of the lowest four exciton states of pyrene dimer; (**c**,**d**) adiabatic excitation energy profiles and CT character of the lowest four exciton states of 2-azapyrene dimer. From TDA-ωB97X-D/def2-SVP calculations on dimers with a 3.4 Å interplanar distance.

The computed excitation energy profiles of the lowest four singlet exciton states of the pyrene and 2-azapyrene dimers with a 3.4 Å interplanar distance are collected in Figure 7. Because the lowest excited states of pyrene and 2-azapyrene are both of the $L_{b}$ type, one might have expected the lowest exciton state to be of the same nature ( $L_{b}$ ). Notably, we already identified, in previous sections, the excimer state of both pyrene and 2-azapyrene as derived from the monomer $L_{a}$ state. In agreement, Figure 7 shows that for small displacements from the eclipsed geometry, for both dimers, the lowest-energy exciton state belongs to $A_{g}$ symmetry, which is therefore derived from the $L_{a}$ monomer state. Figure 7 shows a second excimer state, of $B_{g}$ symmetry, in the region of the eclipsed geometry. This exciton state is derived from the monomer $L_{b}$ state and is also strongly stabilized at the eclipsed geometry, although less than the $\;A_{g}$ state. 

These energy profiles confirm an inversion of the $L_{b}/L_{a}$ states for both pyrene and 2-azapyrene, when the aggregate is formed. Such an inversion has been reported also for other excimers such as naphthalene [55,64,65] and has been ascribed to the larger exciton interaction between the $L_{a}$ states due to their larger transition dipole moment. However, the nature of the excimers is intimately connected with the contributions from CT excitations. Interestingly, as show in Figure 7b,d, the CT character of the less stable $B_{g}$ excimer state is smaller (ca. 0.25) than that of the more stable $A_{g}$ excimer state (ca. 0.45), revealing that the lower stability of the $L_{b}$ -derived excimer state is due to a smaller exciton interaction combined with a smaller CT character for both the pyrene and 2-azapyrene dimers. 

**Figure 8:**
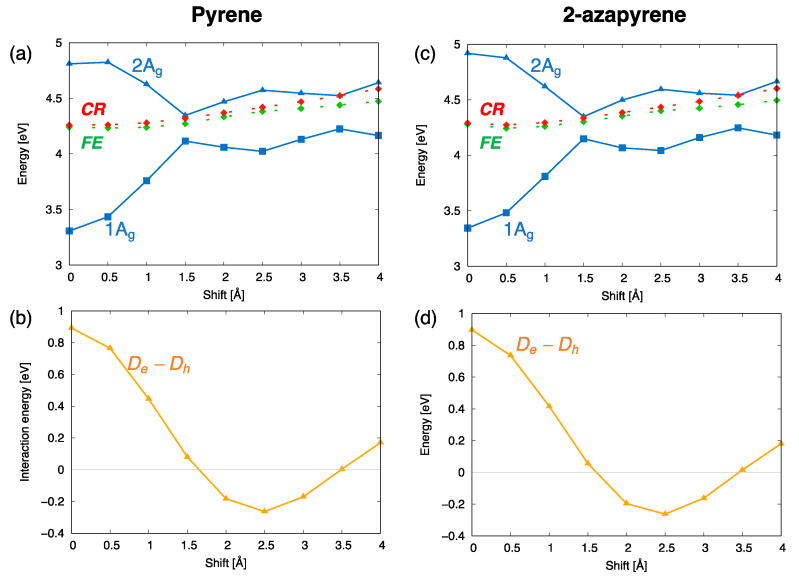
Analysis of the interactions between diabatic states leading to the computed excitation energy profiles of the lowest (adiabatic) exciton states ( $A_{g}$ symmetry) for (**a**,**b**) pyrene and (**c**,**d**) 2-azapyrene dimers (TDA-ωB97X-D/def2-SVP). The investigation is carried out in terms of SA diabatic states (green for FE states, red for CR states) and their interactions. (**a**,**c**) Computed energy profiles of the adiabatic exciton states and of their contributing SA diabatic states. (**b**,**d**) Size of the (orange) $D_{e}-D_{h}$ interactions, coupling FE and CR states along with their evolution along the longitudinal translation coordinate.

The computed trends of the adiabatic energy profiles as a function of the displacement coordinate can be explained by inspecting the interstate interactions between the SA diabatic states. These effects can be appreciated by comparing the energy profiles of the SA diabatic states and the adiabatic energy profiles of $\;A_{g}$ symmetry (Figure 8). Specifically, the formation of the excimer state (the lowest $A_{g}$ state in the figure) results essentially from the interactions between the diabatic CR and  FE states, indicated with red and green dotted lines in the figure. Their interactions (orange curves in Figure 8b,d) display oscillating magnitudes along the translation coordinate, with a maximum value in the eclipsed geometry. Such a strong interaction lowers the adiabatic $A_{g}$ state and stabilizes the excimer with a similar mechanism in pyrene and 2-azapyrene. Thus, the diabatization reveals the crucial role of the CR states, mediated by their strong interactions with local excitations, which stabilize the excimer states in both pyrene and 2-azapyrene.

### 2.5. Rotated Excimer Structures and Interconversion from Eclipsed Excimers

As previously reported [30,31], due to the high symmetry of the eclipsed excimer structures investigated in the previous sections, their oscillator strength is exactly zero, and emission from them is forbidden. This is true for both pyrene and 2-azapyrene. To achieve a non-zero oscillator strength for the transition between the excimer and the ground state, a structure with reduced symmetry is necessary. A simple intermolecular motion that can activate the transition dipole moment is the twisting of one molecule with respect to the other. For pyrene dimers, this was explored in previous investigations, and a minimum corresponding to a twist of ca. 28° was found at the TD-BHLYP/TZVP level, with an energy 12 kcal/mol higher than the eclipsed structure [30]. More recently, two additional structures corresponding to rotations of ca. 50° (ca. 4.6 kcal/mol more stable than the eclipsed) and ca. 80° (less stable than the eclipsed) were found at the TD-CAM-B3LYP/6-31G* + D3 level of theory [31]. 

**Figure 9:**
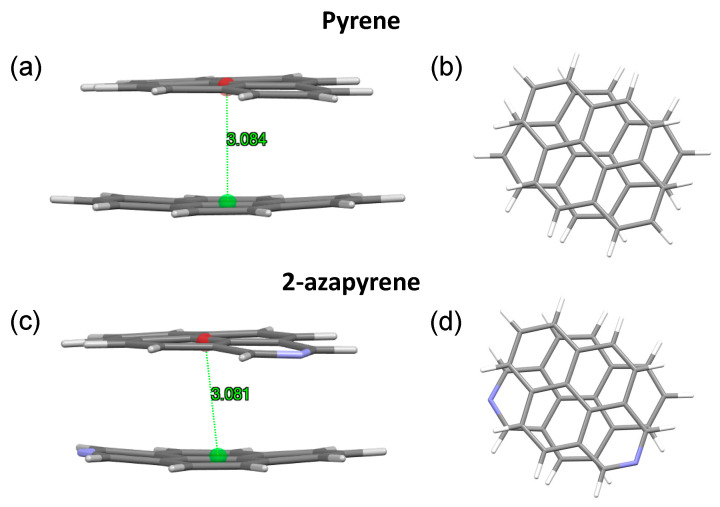
(**a**,**b**) Side and front view of the optimized twisted excimer-state structures, along with intermolecular distances in pyrene and (**c**,**d**) 2-azapyrene (from TD-ωB97X-D/def2-SVP calculations). Intermolecular distances are evaluated between centroids using Mercury [58] over the entire set of atoms of each molecule.

**Figure 10:**
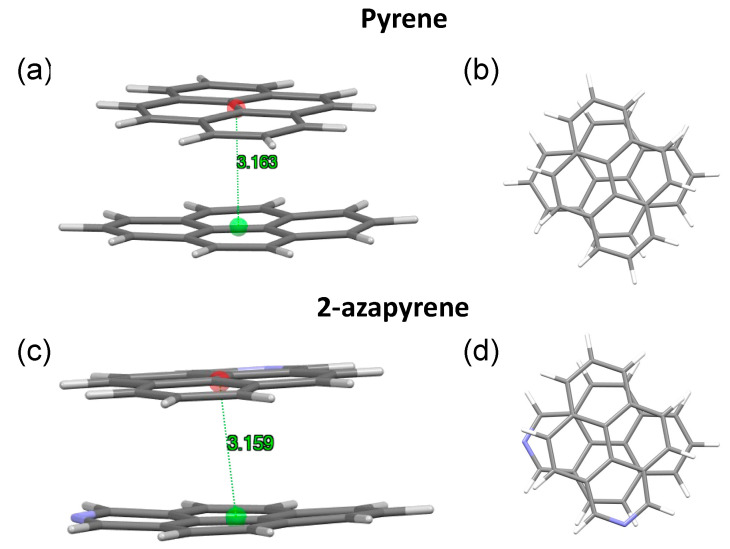
(**a**,**b**) Side and front view of the optimized perp excimer-state structures, along with intermolecular distances in pyrene and (**c**,**d**) 2-azapyrene (from TD-ωB97X-D/def2-SVP calculations). Intermolecular distances are evaluated between centroids using Mercury [58] over the entire set of atoms of each molecule.

We performed a search of the stable rotated structures, via geometry optimization of the lowest exciton state, at the TD-ωB97X-D/def2-SVP level for pyrene and 2-azapyrene and found a similar trend for both dimers. Upon rotation from 0 to 90 degrees, we obtained two equilibrium structures for the lowest exciton state of pyrene and 2-azapyrene. In the first structure (hereafter labeled “twisted”, Figure 9), the two molecules are rotated by ca. 50°, while in the second (labeled “perp”, Figure 10), the two molecules are almost perpendicular to one another and form an angle of ca. 82°. The 50° twisted structures are more stable than the eclipsed by 5.0 kcal/mol for pyrene and 4.5 kcal/mol for 2-azapyrene. The perp structures are found to be less stable than the eclipsed: 5.8 kcal/mol higher for pyrene and 6.6 kcal/mol for 2-azapyrene. 

In both the twisted and perp excimer structures, the molecular units are remarkably bent out of plane. Considering the twisted structures, the 3.084 Å (pyrene) and 3.081 Å (2-azapyrene) distances (Figure 9) between the centroids computed over each monomer represent the average values, with the actual values ranging from 3.02 Å (2.91 Å) to 3.15 Å (3.14 Å) for atoms in the central or in the terminal part of pyrene (2-azapyrene), as can be seen in Figures S5 and S6. Notably, these distances are remarkably reduced compared to the eclipsed excimer. Similar considerations hold for the perp structures (Figures S7 and S8), where the 3.163 Å (pyrene) and 3.159 Å (2-azapyrene) distances between the centroids (Figure 10) can be compared with actual values ranging from 3.09 Å (3.03 Å) to 3.23 Å (3.23 Å) for atoms in the central or in the terminal part of pyrene (2-azapyrene).

Since the twisted excimer structures are remarkably more stable than the eclipsed ones and feature non-zero transition dipole moments (Table S2), these structures are likely to be responsible for the observed pyrene excimer emission. 

**Figure 11:**
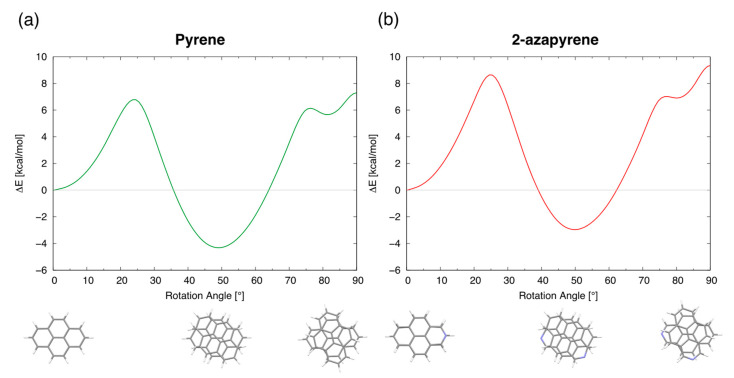
Energy profiles of the lowest exciton state, along the intermolecular rotation coordinate, for (**a**) pyrene and (**b**) 2-azapyrene. The energy profile is determined by rigidly rotating one molecule with respect to the other. The energy of the eclipsed geometry is taken as reference.

To obtain more insight into the conversion from eclipsed into twisted minimum structures, we determined, using model calculations based on rigidly rotated monomers, the energy profile of the lowest singlet exciton state along the intermolecular rotation coordinate. Such an energy profile (Figure 11) shows indeed three minima for the twisting angles in perfect agreement with the three optimized excimer structures described above. Specifically, the computed energy profiles show that the conversion from the eclipsed into the twisted excimer structures (as well as the conversion from the twisted into the perp structures) requires overcoming a barrier which is originated by an avoided crossing, implying a change in wavefunction nature. 

**Figure 12:**
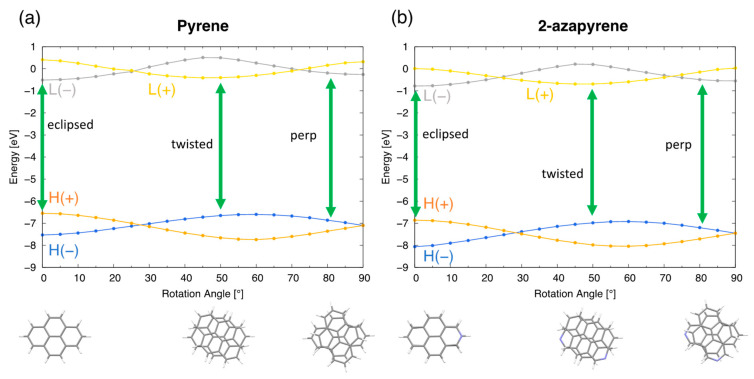
Frontier orbital energy profiles of pyrene (**a**) and 2-azapyrene (**b**) along the twisting coordinate. The orbital excitation dominating each optimized excimer structure (eclipsed, twisted and perpendicular) is also indicated with green arrows. (HOMO and LUMO abbreviated as H and L). Labels (H(+/−) and L(+/−)) for the dimer orbitals indicate their linear combinations of monomer H_A,B_ and L_A,B_ orbitals. See also Figure 4.

The modulation of the frontier molecular orbital energies (Figure 12) along the intermolecular rotation coordinate facilitates the analysis of the wavefunction for the three minima, by keeping in mind that the wavefunction of the three excimer structures is dominated by the HOMO→LUMO excitation. As discussed in previous sections (Figure 4), these frontier orbitals are linear combinations of the monomer orbitals, and the (+/−) label holds valid upon rotation. Abbreviating HOMO and LUMO as H and L , the eclipsed, twisted and perp excimers correspond to the H(+)→L(−), H(−)→L(+) and H(−)→L(−) excitations, respectively, namely to three different orbital natures. In all cases, the excited LUMO orbital acquires a bonding nature (Figure S9) that justifies the stabilization of the excimer structure. 

Although it is commonly understood that, in solution, the formation of an excimer occurs through a diffusion-controlled reaction between an excited singlet molecule and a ground-state molecule [60], in previous studies, it has been suggested that the excimer could be formed via the direct excitation of ground-state dimers [29,71]. Thus, based on the barriers in Figure 11, we can discuss a possible alternative pathway for the formation of excimers in solution: if a ground-state dimer is formed in the parallel displaced structure, the eclipsed excimer structure will be formed in a few ps after excitation, and, eventually, the more stable twisted excimer structure will be reached by overcoming the barrier to rotation (Figure 11) during the lifetime of the excimer (50–100 ns [72,73]). From the data in Figure 11, a barrier of 6.8 kcal/mol is estimated for pyrene and 8.8 kcal/mol for 2-azapyrene, the latter larger by 2 kcal/mol. While additional calculations will be required to estimate more accurately such barriers, the larger barrier of 2-azapyrene is confirmed also by calculations carried out using different monomer geometries. Thus, we are confident that the larger computed barrier of 2-azapyrene reflects its more relevant electrostatic interactions, preferentially stabilizing the eclipsed excimer structure. Assuming a simple Eyring formulation for the rate constant associated with the $∆E^{\#}$ barrier ( $k=\frac{KT}{h}e^{\left(-\frac{∆E^{\#}}{KT}\right)}$ ), at 300 K, we obtain k= 7.5 × 10^7^ s^−1^ and 2.8 × 10^6^ s^−1^ for pyrene and 2-azapyrene, respectively. This implies half lives $t_{1/2}\;$ of about 9 ns for pyrene, which is within the lifetime of the excimer (ca. 50–100 ns in cyclohexane [72,73]), while for 2-azapyrene, a $t_{1/2}$ of about 250 ns could prevent the formation of the more stable and emitting excimer structure through this alternative pathway, although diffusion-controlled formation of the excimer should always be viable. Thus, we can conclude that the role of the dipole–dipole interaction in 2-azapyrene dimers might prevent the activation of this alternative pathway for the formation of a bright excimer and could influence its excimer-emitting properties.

## 3. Computational Details

The ground-state monomer structure of pyrene and 2-azapyrene was optimized at the ωB97X-D/def2-SVP level of theory. Geometry optimization of the ground-state dimers was conducted at the same level of theory, while optimization of the excimer structures was performed at the TD-ωB97X-D/def2-SVP level of theory, by selecting the lowest excited state (keywords: opt and TD(root = 1)). We note that the dispersion interactions described by the ωB97X-D functional might not be optimized for excited states. However, we believe that any eventual unbalanced description is expected to be similar for both molecules/dimers given the similar structural and electronic nature of the excited/excimer states. Furthermore, we note that this functional has been successfully used in the discussion of exciton states of several PAH aggregates [55,67,68,74]. 

The analysis of the interplanar and intermolecular distances was performed using the Mercury software 2021.2.0 [58].

To investigate the nature of the lowest-energy exciton states, excited state calculations were carried out starting from the eclipsed dimer structure and displacing one monomer with respect to the another along the longitudinal translation coordinate by increments of 0.5 Å up to 4.0 Å. The molecules were taken at their ground-state optimized geometry (ωB97X-D/def2-SVP) and the interplanar distance was set to 3.4 or 3.2 Å, namely close to the ground-state dimer structure or excimer-state structure, respectively. The excitation energies were obtained using TD-DFT calculations and using the Tamm–Dancoff approximation (TDA) [75]. We adopted the ωB97X-D functional [76] since it was shown to provide a suitable description of the CT character in the singlet excitons of other PAH dimers [45,55,67,68] and the def2-SVP basis set. All quantum chemical calculations were carried out using Gaussian16 [77]. A comparison between the exciton states computed for 3.4 or 3.2 Å interplanar distances is collected in Figure S10.

The singlet exciton states of pyrene and 2-azapyrene dimers, computed using TDA TD-DFT calculations, were analyzed using a diabatization procedure to determine their character ( CT / LE ) and to unravel the effect of the interactions between diabatic states along the displacement coordinate. According to the MOs depicted in Figure 2, and the orbital nature of the lowest-lying excited states of the monomers, the orbital space selected in the analysis of the exciton states included four MOs for each monomer (two occupied and two unoccupied), and, consequently, a total of eight orbitals for their dimers. Because of the intermolecular interactions, the energy profiles of the dimer orbitals display oscillations along the longitudinal displacement that lead to crossings between the HOMO/HOMO−1, HOMO−2/HOMO−3, LUMO/LUMO + 1 and LUMO + 2/LUMO + 3 orbitals of the dimers (Figures S11 and S12).

In practice, the diabatization expresses the states resulting from the TD-DFT calculations on the dimer in terms of single excitations between monomeric orbitals (the diabatic states), the latter defined as LE or CT *,* as discussed in the introduction section, on the basis of localized orbitals on monomers A and B . To extract the exciton character and the relevant interstate interactions, we used the approach described in our previous works [45,55,67,68]. Essentially, to obtain each relevant exciton state in terms of the diabatic states, we first express each aggregate orbital as a linear combination of monomer orbitals [67,78,79]. Then, we transform the diagonal Hamiltonian $H_{adia}$ matrix, formed by the eigenvalues of the selected adiabatic exciton states, into the $H_{dia}$ matrix [39,48,50,80], representing the Hamiltonian in the diabatic LE / CT basis, as detailed in the Supporting Information section. Finally, the $H_{dia}$ is transformed into the $H_{dia}^{SA}\;$ matrix, a representation of the Hamiltonian in the SA diabatic basis formed by the FE and CR states. This matrix can be recast into a block diagonal form composed by sub-matrices for different symmetries (corresponding to $B_{u}$ , $A_{g},$ $B_{g}$ and $A_{u}$ states: see Tables S6 and S7). The off-diagonal elements in each sub-matrix represent the interactions between CR and FE states (including excitonic interactions $V_{e}^{\left(n\right)}$ and super-exchange interactions [81] $D_{e/h}$ ) that control the modulation of the adiabatic exciton-state energies along the displacement coordinate. Thus, this analysis discloses the intermolecular interactions leading ultimately to character and energy modulation of the exciton states. 

Finally, to determine the energy barriers for conversion from the eclipsed into the twisted excimers, the energy profile of the lowest singlet exciton state under the rotation of one molecule with respect to the other was determined by rigidly rotating one molecule with respect to the other by 5° from the eclipsed to the perpendicular configuration. The molecules were kept at their ground state optimized geometry (ωB97X-D/def2-SVP) and the interplanar distance was set to 3.2 Å, which is close to the intermolecular distances for the excimer structures. 

## 4. Conclusions

In this work, we have investigated pyrene and 2-azapyrene dimers to uncover the similarities and differences responsible for their photophysical properties, with a focus on the excimer emission properties. 

A strong indication of the formation of excimers in both pyrene and 2-azapyrene dimers comes from an analysis of the exciton energy profiles and associated wavefunctions along the longitudinal translation coordinate. The diabatization reveals, around the eclipsed geometry, a remarkable stabilization of the lowest $A_{g}$ exciton state (derived from the $L_{a}$ excited state of the monomers), driven by the strong mixing with CR excitations, favored by large FE / CR interstate interactions. A second excimer state (derived from the $L_{b}$ state of the monomers), also stabilized at the eclipsed geometry, is disclosed for both the pyrene and 2-azapyrene dimers. Its lower stabilization can be rationalized by the combination of smaller exciton interactions and smaller CT (CR)  contributions. Thus, such different CT contributions drive the $L_{a}$ / $L_{b}$ state inversion of the lowest-lying exciton state of the pyrene and 2-azapyrene dimers. 

This study shows that the exciton states and excimer formation in the pyrene and 2-azapyrene dimers follow a similar trend, due to their comparable electronic structures. This is confirmed by the full geometry optimization of the excimer structures, which are very similar for the two PAH derivatives. However, a major effect of nitrogen substitution is the appearance of a non-negligible dipole moment in the monomer of 2-azapyrene, which impacts the stabilization of the different excimer structures (eclipsed, twisted and perp), identified using geometry optimization, and, more importantly, their conversion barriers. 

Our study also shows that although all three excimer structures are derived from the same $L_{a}$ excited state of the monomer, their wavefunction nature changes from a dark eclipsed structure into a more stable and bright twisted structure, and similarly when moving to the higher-energy perp structure. The change in wavefunction nature is associated with an energy barrier, for the conversion from eclipsed into twisted, larger by ca. 2 kcal/mol for 2-azapyrene, which might influence the excimer emission properties of the nitrogen-substituted derivative.

Understanding the photophysics of molecular π-stacked chromophores is fundamental to exploiting their properties in functional photonic materials. In this sense, this work, by analyzing deeply the nature of the exciton/excimer states in pyrene and its nitrogen-substituted derivative, as well as the possible reasons for their different photophysical behavior, paves the way for understanding the photoinduced properties of more complex molecular organizations in aggregates and crystalline structures. 

## Data Availability

The data presented in this study are available in the Supplementary Materials.

## Supplementary Materials

The following supporting information can be downloaded at www.mdpi.com/xxx/s1: Figures S1–S12, Tables S1–S7, Cartesian coordinates of the optimized structures.
