# Supplementary material for: Eclipsed and Twisted Excimers of Pyrene and 2-Azapyrene: How Nitrogen Substitution Impacts Excimer Emission
Source: Molecules. 2024 Jan 19;29(2):0. doi: 10.3390/molecules29020507 (PMC11154402; doi:10.3390/molecules29020507)
Supplement: Supplementary file 1 [file molecules-29-00507-s001.zip › molecules-2821075-supplementary.pdf]

Supporting Information for

Eclipsed and twisted excimers of pyrene and 2-azapyrene: how nitrogen substitution impacts excimer emission

Yasi Dai [1,2], Filippo Rambaldi[1] and Fabrizia Negri [1,2,3*]

[1]Dipartimento di Chimica “Giacomo Ciamician”, Università di Bologna, 40126 Bologna, Italy

[2]Center for Chemical Catalysis – C3, Alma Mater Studiorum – Università di Bologna, Via Selmi 2, 40126 Bologna, Italy

[3]INSTM UdR Bologna, Italy

*Correspondence: fabrizia.negri@unibo.it

Computational details: diabatization

The number of spin-adapted singly excited configurations (and states) originated by an orbital space of n doubly occupied and n unoccupied orbitals is n^2, such that sixteen exciton states have been considered for each spin multiplicity and their wavefunctions expressed in terms of diabatic states, with the diabatization procedure outlined in Section 3. The protocol employed to analyze the intermolecular interactions leading ultimately to the character of exciton states, transforms the amplitudes of TDDFT calculations carried out on the aggregate, from the basis of single excitations between aggregate's orbitals (the delocalized excitation (DE) basis) to the basis of single excitations between molecular (monomers A and B) site orbitals. Localized orbitals on monomers A and B allow to define diabatic states of four different types as follows:

$$
\begin{aligned}
LE_A^{(1)} &= |1_A\rangle = (H-1)_A \rightarrow (L+1)_A \\
LE_B^{(1)} &= |1_B\rangle = (H-1)_B \rightarrow (L+1)_B \\
CT_{AB}^{(1)} &= |1_{AB}\rangle = (H-1)_A \rightarrow (L+1)_B \\
CT_{BA}^{(1)} &= |1_{BA}\rangle = (H-1)_B \rightarrow (L+1)_A \\
\\
LE_A^{(2)} &= |2_A\rangle = (H-1)_A \rightarrow (L)_A \\
LE_B^{(2)} &= |2_B\rangle = (H-1)_B \rightarrow (L)_B \\
CT_{AB}^{(2)} &= |2_{AB}\rangle = (H-1)_A \rightarrow (L)_B \\
CT_{BA}^{(2)} &= |2_{BA}\rangle = (H-1)_B \rightarrow (L)_A \\
\\
LE_A^{(3)} &= |3_A\rangle = (H)_A \rightarrow (L)_A \\
LE_B^{(3)} &= |3_B\rangle = (H)_B \rightarrow (L)_B \\
CT_{AB}^{(3)} &= |3_{AB}\rangle = (H)_A \rightarrow (L)_B \\
CT_{BA}^{(3)} &= |3_{BA}\rangle = (H)_B \rightarrow (L)_A \\
\\
LE_A^{(4)} &= |4_A\rangle = (H)_A \rightarrow (L+1)_A \\
LE_B^{(4)} &= |4_B\rangle = (H)_B \rightarrow (L+1)_B \\
CT_{AB}^{(4)} &= |4_{AB}\rangle = (H)_A \rightarrow (L+1)_B \\
CT_{BA}^{(4)} &= |4_{BA}\rangle = (H)_B \rightarrow (L+1)_A
\end{aligned}
\tag{1}
$$

where HOMO and LUMO are abbreviated as H and L, subscripts A and B indicate different monomers, and each type of excitation is differentiated by a superscript.

The diabatization procedure provides the Hamiltonian matrix in the diabatic basis representation, $\boldsymbol{H}_{dia}$. In this work the $\boldsymbol{H}_{dia}$ matrix (dimension 16x16) includes not only interactions between diabatic states of the same type ($LE_A^{(n)}, LE_B^{(n)}, CT_{AB}^{(n)}, CT_{BA}^{(n)}$), such as excitonic interactions $V_e^{(n)}$ and super-exchange interactions [1] $D_{e/h}^{(n)}$ (Table S3), but also interactions between diabatic states of different type ($LE/CT^{(n)}, LE/CT^{(p)}$), hereafter labeled with two superscript numbers, e.g., $V_e^{(n,p)}$ (Table S4). In addition, intramolecular interactions between diabatic states localized on the same monomer

($LE_A^{(n)}, LE_A^{(p)}$) are also uncovered by the diabatization procedure ($H^{(n,p)}$ in Table S4). In the following Figures, each symmetry of resulting CR or FE symmetry adapted (SA) diabatic states, will be distinguished by a specific color code: blue for A_g, orange for A_u, green for B_g and yellow for B_u.

The SA diabatic states are then obtained as linear combinations of LE and CT states as shown in Table S5 and the corresponding diabatic matrices $\boldsymbol{H}_{dia}^{SA}$ are collected in Tables S6 and S7.

To analyze the exciton character, we followed the approach described in previous works [2,3] and we expressed each relevant exciton state in terms of LEs. To this end, we selected the orbital subspace corresponding to relevant $\pi\pi^*$ exciton states. This represents the minimal orbital space (MIOS) sufficient to reliably describe low-lying excited states of each monomer. Each aggregate's orbital obtained from quantum-chemical calculations is then expressed as linear combination of monomer orbitals. These linear combination coefficients $C_{i,j}^{AGGR\_MOB}$ form the $\boldsymbol{C}_{AGGR\_MOB}$ matrix describing each aggregate's orbital in the monomer orbital basis (MOB) and are obtained as [2,4,5]:

$$\boldsymbol{C}_{AGGR\_MOB} = \boldsymbol{C}_{MON\_AOB}^{t} \cdot \boldsymbol{S}_{MON\_AOB} \cdot \boldsymbol{C}_{AGGR\_AOB} \tag{2}$$

where the $\boldsymbol{C}_{MON\_AOB}$ matrix is a block diagonal matrix containing the MOs coefficients in the atomic orbital basis (AOB) of each monomer, with off-diagonal blocks set to zero and $\boldsymbol{S}_{MON\_AOB}$ is the overlap matrix of the monomers in the AOB.

Since monomer orbitals belonging to two different molecules are non-orthogonal to each other, aggregate's orbitals $\boldsymbol{C}_{AGGR\_MOB}^{L}$ expressed in terms of orthogonalized monomer orbitals are obtained as:

$$\boldsymbol{C}_{AGGR\_MOB}^{L} = \boldsymbol{S}_{AGGR\_MOB}^{-\frac{1}{2}} \cdot \boldsymbol{C}_{AGGR\_MOB} \tag{3}$$

where superscript L indicates Löwdin's orthogonalization [6], and the overlap matrix $\boldsymbol{S}_{AGGR\_MOB} = \boldsymbol{C}_{MON\_AOB}^{t} \cdot \boldsymbol{S}_{AGGR\_AOB} \cdot \boldsymbol{C}_{MON\_AOB}$ is obtained from the coefficients of monomer's orbitals $\boldsymbol{C}_{MON\_AOB}$ and the overlap of the atomic orbitals in the aggregate configuration $\boldsymbol{S}_{AGGR\_AOB}$.

From the results of TDDFT calculations on the aggregate, the subset of n^2 exciton states originated from the MIOS of the aggregate are then selected out of the full set of computed eigenstates. TDDFT amplitudes are expressed on the basis of delocalized excitations (DEs), namely excitations between aggregate's orbitals, and form the columns of the $\boldsymbol{B}_{DE}^{adia}$ matrix. Thus, each DE must be expanded in terms of excitations between monomer orbitals (diabatic LE and CT states). With aggregate's orbitals expressed in terms of monomer orbitals via the $\boldsymbol{C}_{AGGR\_MOB}^{L}$ matrix, each DE($i \rightarrow j$) from an occupied *i* to an empty *j* aggregate's orbital can be expressed as a linear combination of diabatic (LE and CT) excitations ($k \rightarrow l$) from an occupied *k* to an empty *l* monomer orbital, with expansion coefficients given by

$$U_{k\rightarrow l,i\rightarrow j}^{DE\rightarrow dia} = C_{k,i}^{AGGR\_MOB,L} \cdot C_{l,j}^{AGGR\_MOB,L} \tag{4}$$

these coefficients form the columns of the unitary matrix $\boldsymbol{U}_{\boldsymbol{DE}\rightarrow\boldsymbol{dia}}$.
Exciton states are then readily expressed in the diabatic basis as

$$\boldsymbol{B}_{dia}^{adia} = \boldsymbol{U}_{\boldsymbol{DE}\rightarrow\boldsymbol{dia}} \cdot \boldsymbol{B}_{DE}^{adia} \quad (5)$$

and the character of each exciton state is obtained by summing up the contributions from CT and LE states.
The corresponding $\boldsymbol{n}^{\boldsymbol{2}}$ eigenvalues (excitation energies of the selected adiabatic excitons) form the diagonal $\boldsymbol{H}_{adia}$ matrix, from which the Hamiltonian in the diabatic LE/CT basis, $\boldsymbol{H}_{dia}$, can be obtained as [7–10]

$$\boldsymbol{H}_{dia} = \boldsymbol{B}_{dia}^{adia} \cdot \boldsymbol{H}_{adia} \cdot {\boldsymbol{B}_{dia}^{adia}}^{t} \quad (6)$$

Table S1. Lowest-lying singlet and triplet excited states of Pyrene and 2-azapyrene molecules. Calculations performed at TD-ωB97X-D/def2-SVP level of theory. The Platt's notation for each excited state with the appropriate orbital nature is also indicated.

**Pyrene**						
State	Sym.	Energy (eV)	λ (nm)	Osc. strength	Wavefunction	Exp. (eV)[a]
S_1 (L_b)	B2U	4.03	308	0.0002	0.48 H-1→L<br>0.51 H→L+1	3.34
S_2 (L_a)	B1U	4.07	304	0.3277	-0.23 H-1→L+1<br>0.67 H→L	3.70
T_1 (L_a)	B1U	2.08	595	0	0.67 H→L	
T_2	B3G	3.64	340	0	0.45 H-2→L<br>0.49 H→L+2	
T_3 (L_b)	B2U	3.75	331	0	-0.38 H-1→L<br>0.57 H→L+1	
**2-azapyrene**						
State	Sym.	Energy (eV)	λ (nm)	Osc. strength	Wavefunction	Exp. (eV)[b]
S_1 (L_b)	B2	4.05	306	0.0211	0.38 H-1→L<br>0.59 H→L+1	3.31
S_2 (L_a)	A1	4.10	303	0.3430	-0.22 H-1→L+1<br>0.67 H→L	3.35
T_1 (L_a)	A1	2.09	593	0	0.67 H→L	
T_2 (L_b)	B2	3.55	349	0	0.66 H→L+1	

[a] Experimental results from absorption spectrum in iso-octane (Ref. [11])
[b] Experimental results from absorption spectrum in ethanol (Ref. [12])

Table S2. Emission energy and oscillator strength of the three optimized excimer structures of Pyrene and 2-azapyrene dimers. Calculations performed at TD-ωB97X-D/def2-SVP level of theory.

**Pyrene excimer**				
	Emission energy / eV	Emission energy / nm	f	Experimental value / nm
eclipsed	2.55	487	0.000	
twisted	2.43	511	0.018	478
perp	3.04	407	0.070	
**2azapyrene excimer**				
	Emission energy / eV	Emission energy / nm	f	Experimental value / nm
eclipsed	2.48	499	0.000	
twisted	2.38	521	0.018	–
perp	3.01	411	0.071	

Table S3. Matrix elements of $\boldsymbol{H}_{dia}$ between diabatic states of the same type $(LE^{(n)}, CT^{(n)})$.

$\boldsymbol{H}_{dia}$	$LE_A^{(n)}$	$LE_B^{(n)}$	$CT_{AB}^{(n)}$	$CT_{BA}^{(n)}$
$LE_A^{(n)}$	$E_{LE}^{(n)}$	$V_e^{(n)}$	$D_e^{(n)}$	$D_h^{(n)}$
$LE_B^{(n)}$	$V_e^{(n)}$	$E_{LE}^{(n)}$	$D_h^{(n)}$	$D_e^{(n)}$
$CT_{AB}^{(n)}$	$D_e^{(n)}$	$D_h^{(n)}$	$E_{CT}^{(n)}$	$W^{(n)}$
$CT_{BA}^{(n)}$	$D_h^{(n)}$	$D_e^{(n)}$	$W^{(n)}$	$E_{CT}^{(n)}$

Table S4. Matrix elements of $\mathbf{H}_{\mathrm{dia}}$ between diabatic states of different types $(LE^{(n)}, CT^{(n)}$and $LE^{(p)}, CT^{(p)})$. The additional superscript $-p/-n$ for super-exchange interactions denotes the type of LE diabatic state.

$\boldsymbol{H}_{dia}$	$LE_A^{(p)}$	$LE_B^{(p)}$	$CT_{AB}^{(p)}$	$CT_{BA}^{(p)}$
$LE_A^{(n)}$	$H^{(n,p)}$	$V_e^{(n,p)}$	$D_e^{(n,p)-n}$	$D_h^{(n,p)-n}$
$LE_B^{(n)}$	$V_e^{(n,p)}$	$H^{(n,p)}$	$D_h^{(n,p)-n}$	$D_e^{(n,p)-n}$
$CT_{AB}^{(n)}$	$D_e^{(n,p)-p}$	$D_h^{(n,p)-p}$	$H_{CT}^{(n,p)}$	$W^{(n,p)}$
$CT_{BA}^{(n)}$	$D_h^{(n,p)-p}$	$D_e^{(n,p)-p}$	$W^{(n,p)}$	$H_{CT}^{(n,p)}$

Table S5. Definition of SA diabatic states (FE, CR, each symmetry with a specific color code used throughout this work: blue for A_g, orange for A_u, green for B_g and yellow for B_u) defined as linear combinations of diabatic states (black, LE, CT) and employed to analyze the nature and the effect of interactions on the adiabatic exciton states of pyrene and 2-azapyrene dimers.

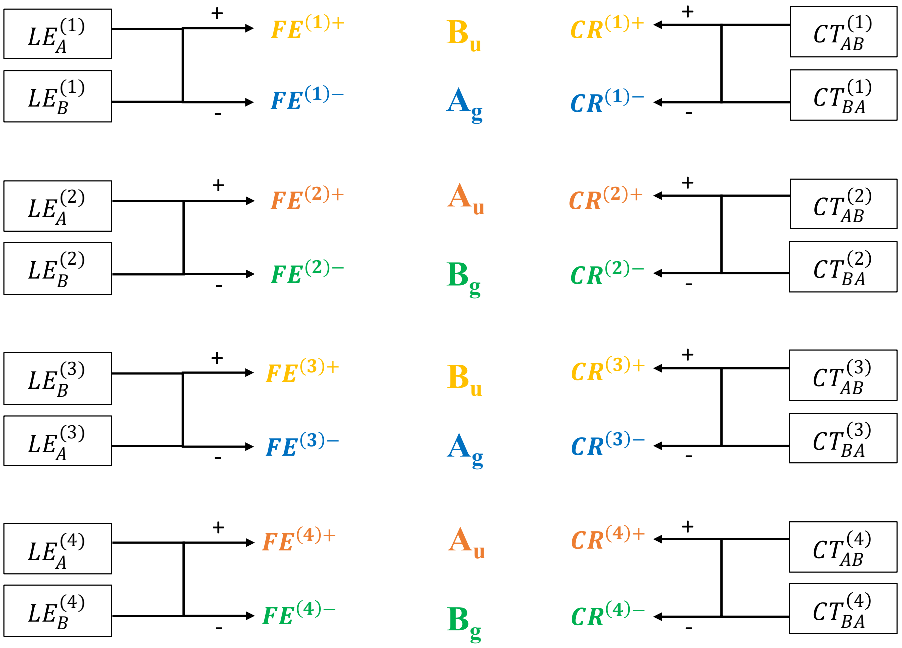

Table S6. Matrix elements of $\boldsymbol{H}_{dia}^{SA}$ between SA diabatic states of $\boldsymbol{A_g}$ symmetry and $\boldsymbol{B_u}$ symmetry).

$\boldsymbol{H}_{dia}^{SA}$ $\boldsymbol{A_g/B_u}$	$FE^{(1)\pm}$	$FE^{(3)\pm}$	$CR^{(1)\pm}$	$CR^{(3)\pm}$
$FE^{(1)\pm}$	$E(LE)^{(1)} \pm V_e^{(1)}$	$H^{(1,3)} \pm V_e^{(1,3)}$	$D_e^{(1)} \pm D_h^{(1)}$	$D_e^{(1,3)-1} \pm D_h^{(1,3)-1}$
$FE^{(3)\pm}$	$H^{(1,3)} \pm V_e^{(1,3)}$	$E(LE)^{(3)} \pm V_e^{(3)}$	$D_e^{(1,3)-3} \pm D_h^{(1,3)-3}$	$D_e^{(3)} \pm D_h^{(3)}$
$CR^{(1)\pm}$	$D_e^{(1)} \pm D_h^{(1)}$	$D_e^{(1,3)-3} \pm D_h^{(1,3)-3}$	$E(CT)^{(1)} \pm W^{(1)}$	$H_{CT}^{(1,3)} \pm W^{(1,3)}$
$CR^{(3)\pm}$	$D_e^{(1,3)-1} \pm D_h^{(1,3)-1}$	$D_e^{(3)} \pm D_h^{(3)}$	$H_{CT}^{(1,3)} \pm W^{(1,3)}$	$E(CT)^{(3)} \pm W^{(3)}$

Table S7. Matrix elements of $\boldsymbol{H}_{dia}^{SA}$ between SA diabatic states of $\boldsymbol{B_g}$ symmetry and $\boldsymbol{A_u}$ symmetry

$\boldsymbol{H}_{dia}^{SA}$ $\boldsymbol{B_g/A_u}$	$FE^{(2)\pm}$	$FE^{(4)\pm}$	$CR^{(2)\pm}$	$CR^{(4)\pm}$
$FE^{(2)\pm}$	$E(LE)^{(2)} \pm V_e^{(2)}$	$H^{(2,4)} \pm V_e^{(2,4)}$	$D_e^{(2)} \pm D_h^{(2)}$	$D_e^{(2,4)-2} \pm D_h^{(2,4)-2}$
$FE^{(4)\pm}$	$H^{(2,4)} \pm V_e^{(2,4)}$	$E(LE)^{(4)} \pm V_e^{(4)}$	$D_e^{(2,4)-4} \pm D_h^{(2,4)-4}$	$D_e^{(4)} \pm D_h^{(4)}$
$CR^{(2)\pm}$	$D_e^{(2)} \pm D_h^{(2)}$	$D_e^{(2,4)-4} \pm D_h^{(2,4)-4}$	$E(CT)^{(2)} \pm W^{(2)}$	$H_{CT}^{(2,4)} \pm W^{(2,4)}$
$CR^{(4)\pm}$	$D_e^{(2,4)-2} \pm D_h^{(2,4)-2}$	$D_e^{(4)} \pm D_h^{(4)}$	$H_{CT}^{(2,4)} \pm W^{(2,4)}$	$E(CT)^{(4)} \pm W^{(4)}$

Pyrene: ground state dimer

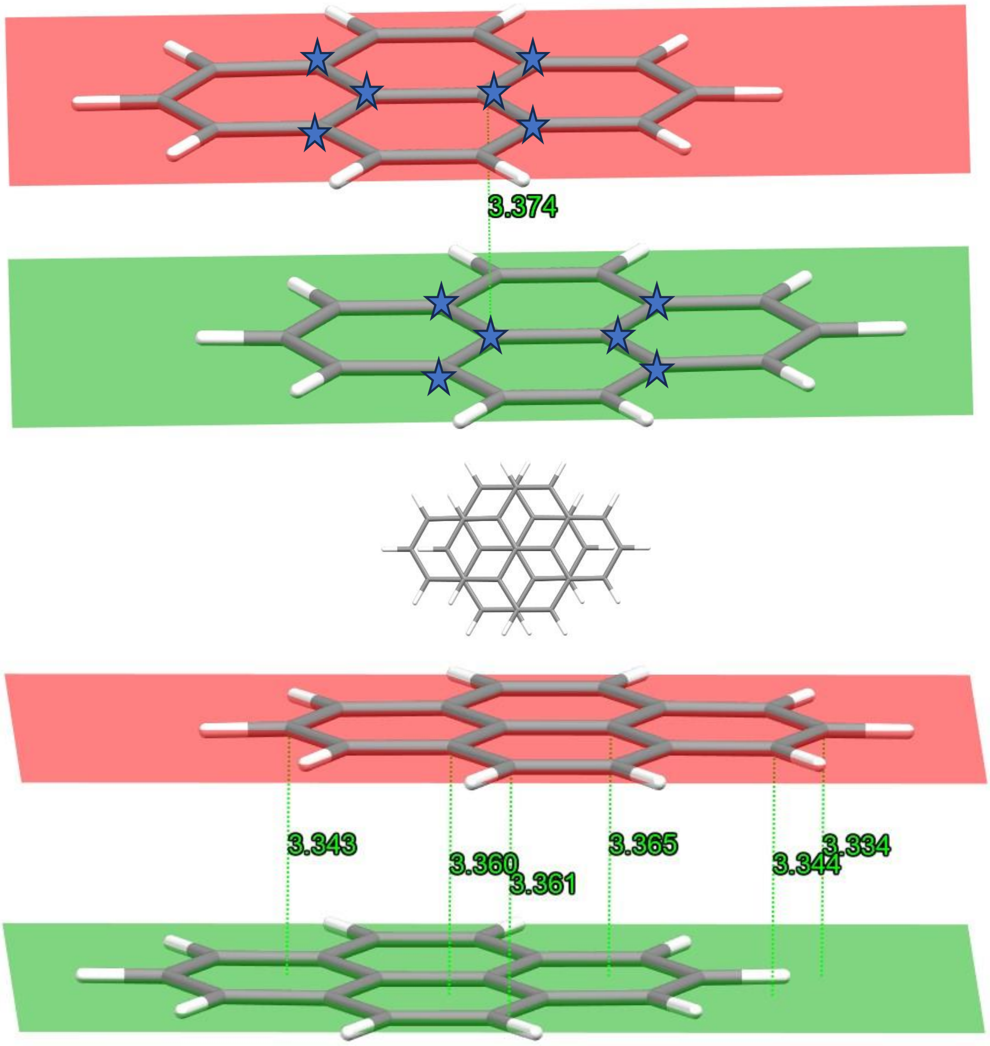

Figure S1. Optimized structure of the ground state of pyrene from ωB97X-D/def2-SVP calculations. (top) Interplanar distance computed between planes defined over the six central carbon atoms indicated by stars. (bottom) Distances of selected atoms, from the central and terminal parts of the monomer on the top, projected on the plane defined over all 26 atoms of the bottom molecule.

2-azapyrene: ground state dimer

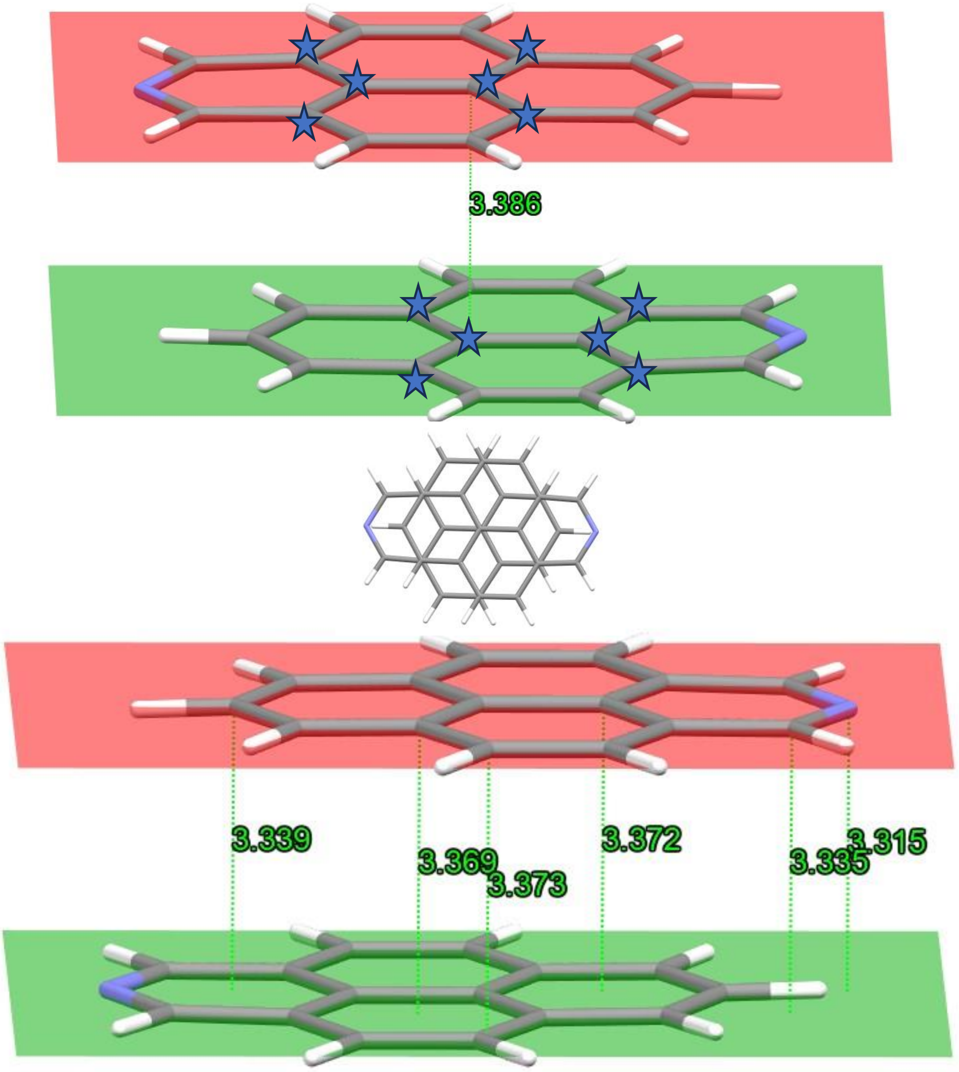

Figure S2. Optimized structure of the ground state of 2-azapyrene from ωB97X-D/def2-SVP calculations. (top) Interplanar distance computed between planes defined over the six central carbon atoms indicated by stars. (bottom) Distances of selected atoms, from the central and terminal parts of the monomer on the top, projected on the plane defined over all 25 atoms of the bottom molecule.

Pyrene: eclipsed excimer

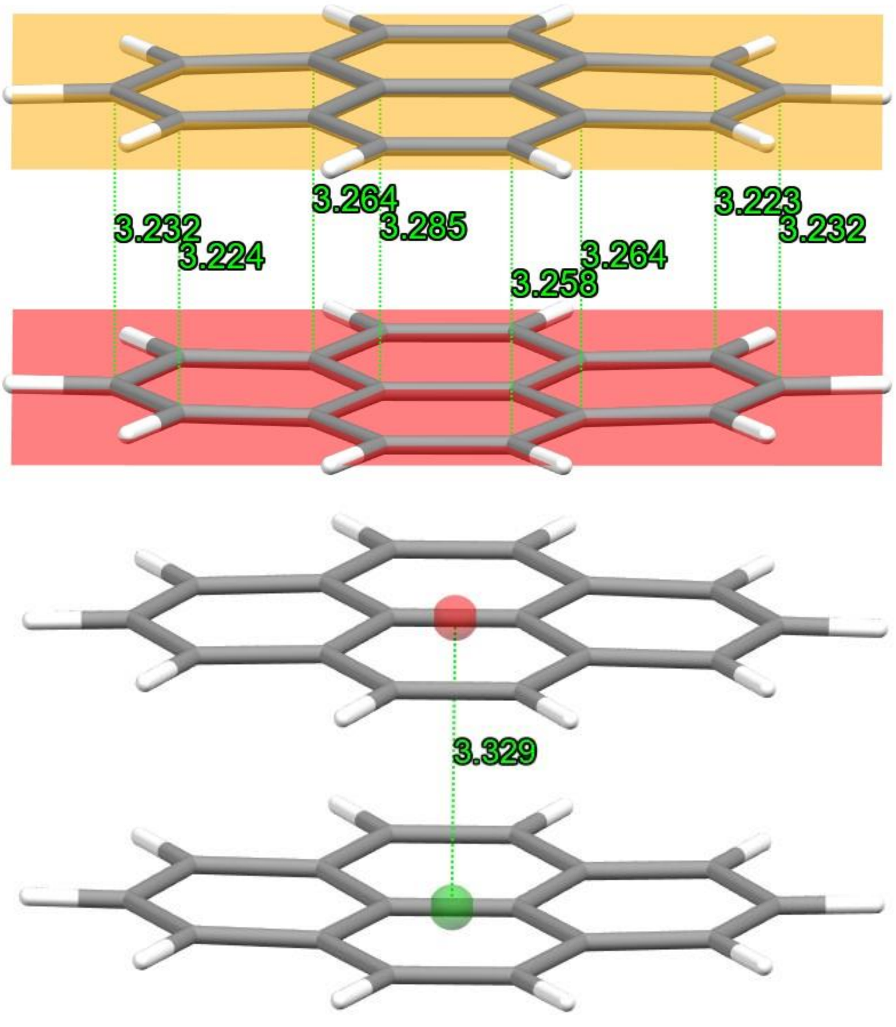

Figure S3. Optimized structure of eclipsed excimer state of pyrene from TD-ωB97X-D/def2-SVP calculations. (top) Distances of selected atoms, from the central and terminal parts of the monomer on the top of the dimer, projected on the plane defined over all 26 atoms of the bottom molecule. (right) Distance between centroids, each determined over the two atoms of the central CC bond of each monomer.

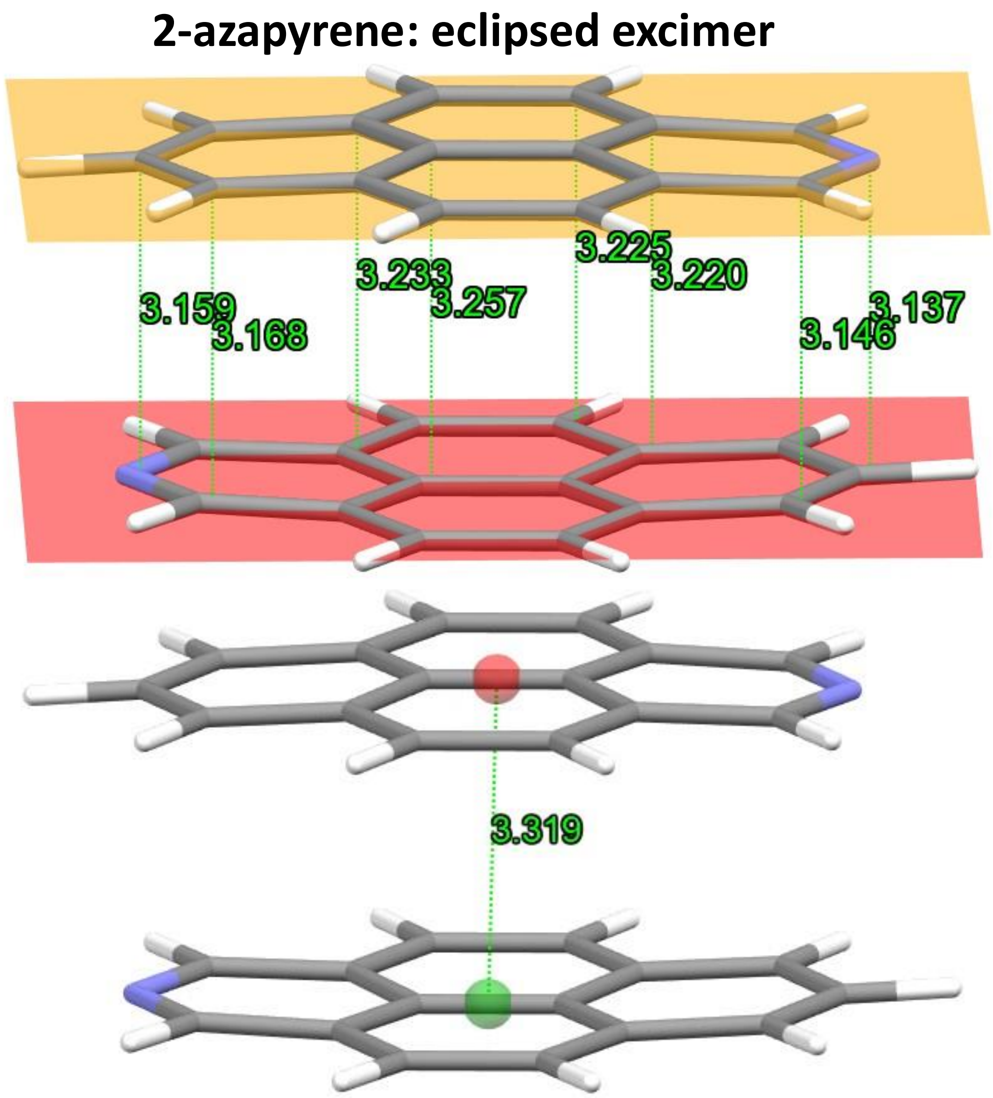

Figure S4. Optimized structure of eclipsed excimer state of 2azapyrene from TD-ωB97X-D/def2-SVP calculations. (top) Distances of selected atoms, from the central and terminal parts of the monomer on the top of the dimer, projected on the plane defined over all 25 atoms of the bottom molecule. (right) Distance between centroids, each determined over the two atoms of the central CC bond of each monomer.

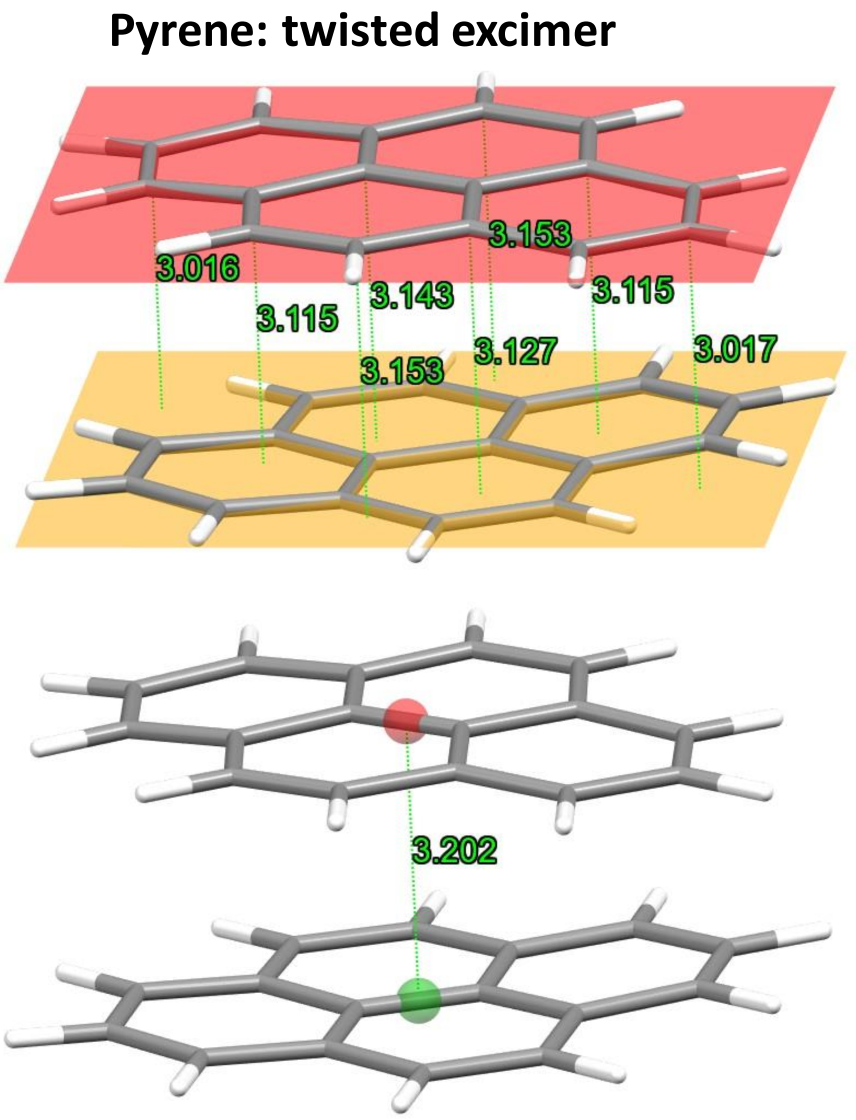

Figure S5. Optimized structure of twisted excimer state of pyrene from TD-ωB97X-D/def2-SVP calculations. (top) Distances of selected atoms, from the central and terminal parts of the monomer on the top of the dimer, projected on the plane defined over all 26 atoms of the bottom molecule. (right) Distance between centroids, each determined over the two atoms of the central CC bond of each monomer.

2-azapyrene: twisted excimer

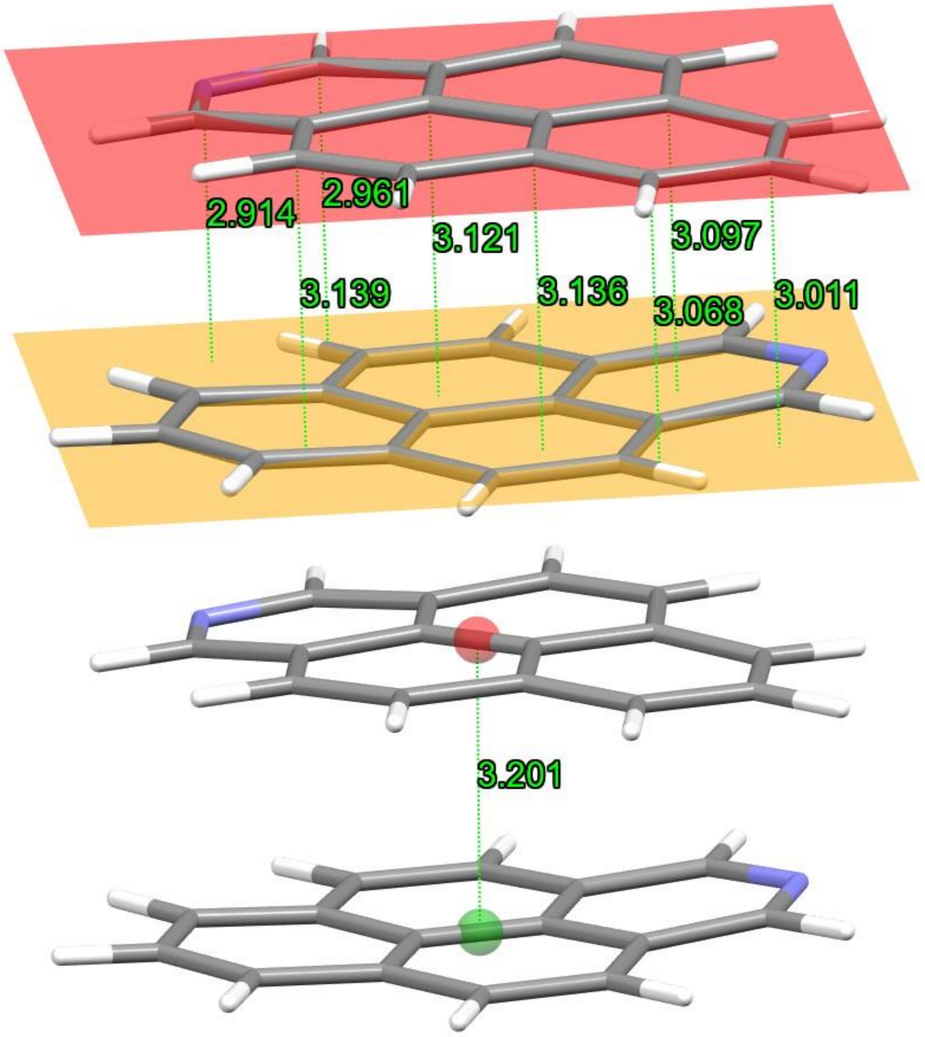

Figure S6. Optimized structure of twisted excimer state of 2azapyrene from TD-ωB97X-D/def2-SVP calculations. (top) Distances of selected atoms, from the central and terminal parts of the monomer on the top of the dimer, projected on the plane defined over all 26 atoms of the bottom molecule. (right) Distance between centroids, each determined over the two atoms of the central CC bond of each monomer.

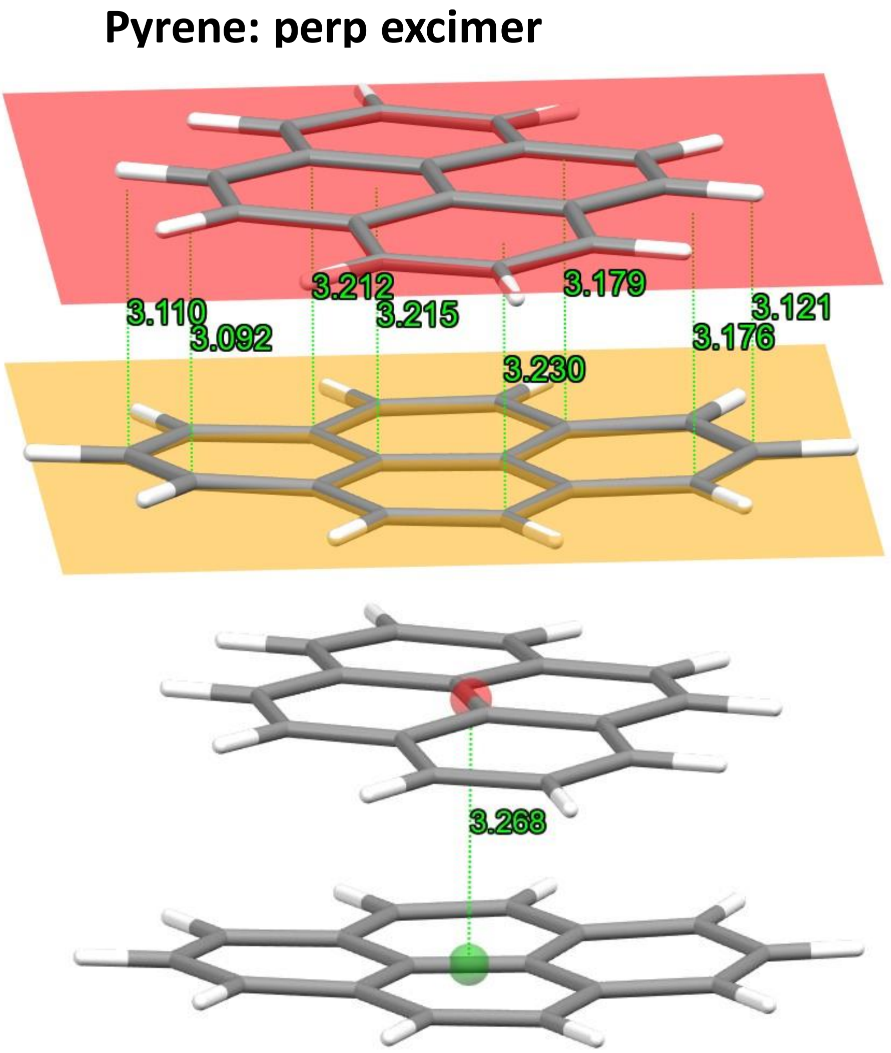

Figure S7. Optimized structure of perp excimer state of pyrene from TD-ωB97X-D/def2-SVP calculations. (top) Distances of selected atoms, from the central and terminal parts of the monomer on the bottom of the dimer, projected on the plane defined over all 26 atoms of the top molecule. (right) Distance between centroids, each determined over the two atoms of the central CC bond of each monomer.

2azapyrene: perp excimer

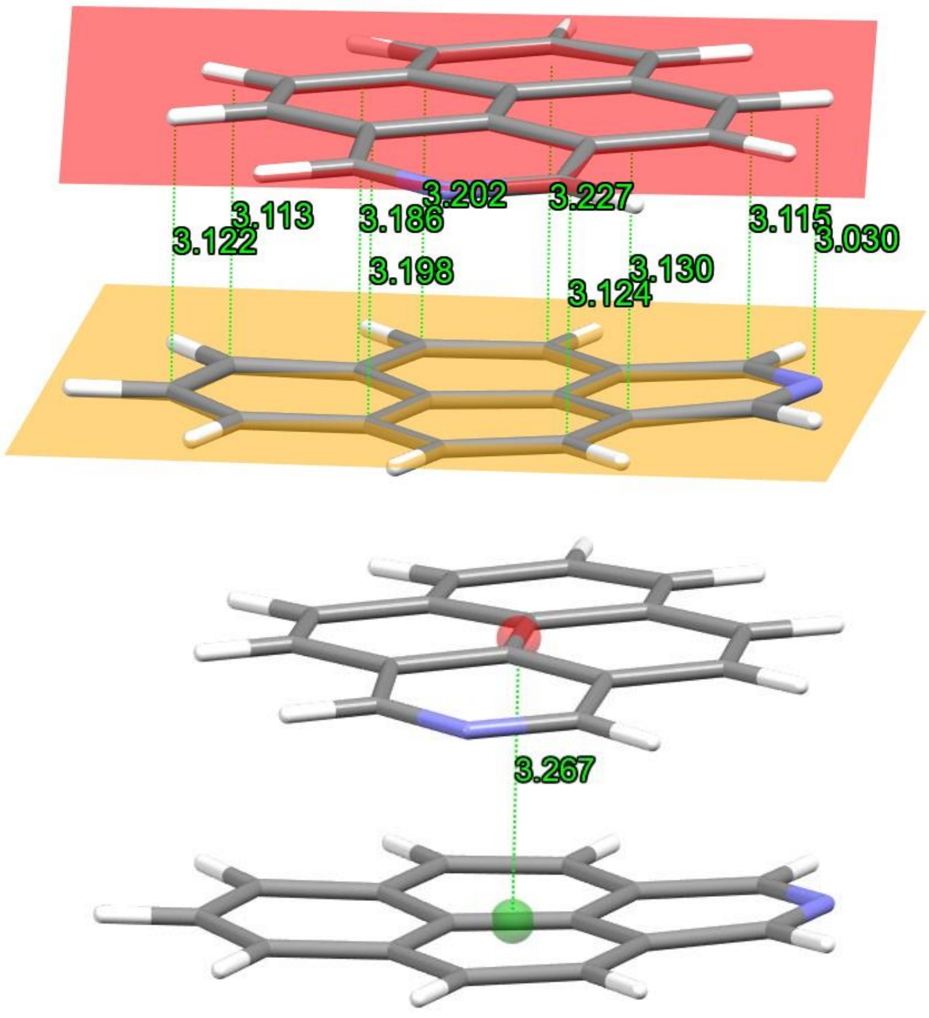

Figure S8. Optimized structure of perp excimer state of 2azapyrene from TD-ωB97X-D/def2-SVP calculations. (top) Distances of selected atoms, from the central and terminal parts of the monomer on the bottom of the dimer, projected on the plane defined over all 26 atoms of the top molecule. (right) Distance between centroids, each determined over the two atoms of the central CC bond of each monomer.

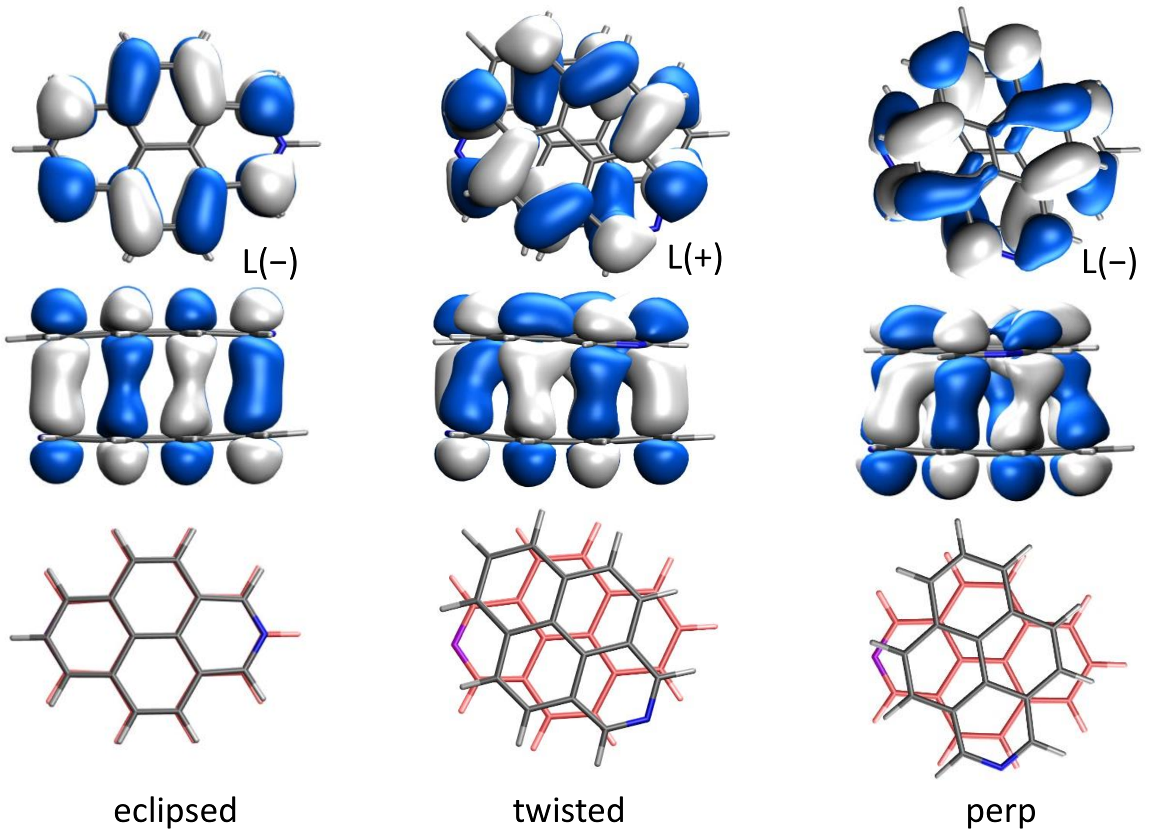

Figure S9. Front and side views of the LUMO (abbreviated as L) for the three optimized excimer structures of 2-azapyrene, from TD-ωB97X-D/def2-SVP calculations. The label (+/−) indicates the appropriate linear combination of LUMO monomer orbitals.

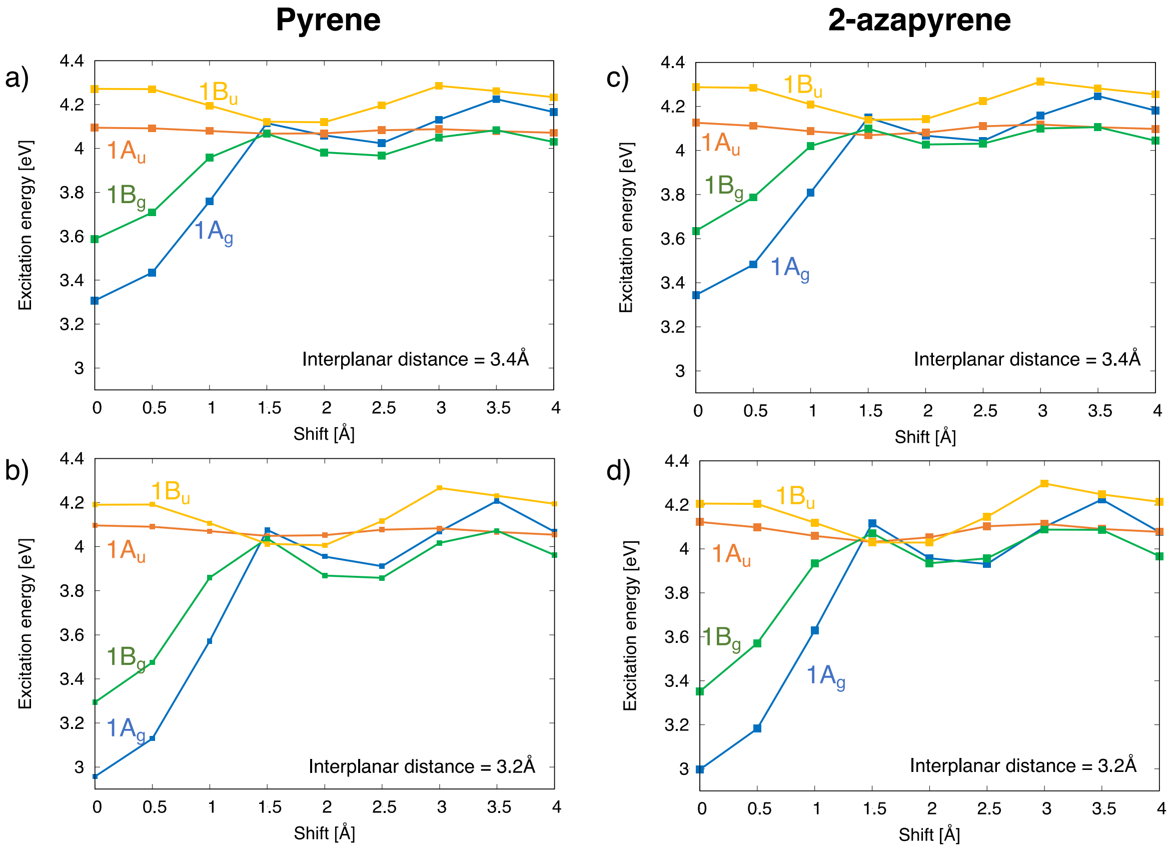

Figure S10. Excitation energy profiles of the four lowest-lying singlet exciton states of (a,b) pyrene and (c,d) 2-azapyrene dimers along the longitudinal shift. (a,c) The interplanar distance considered here is 3.4Å; (b,d) the interplanar distance considered is 3.2Å (from TDA-ωB97X-D/def2-SVP calculations).

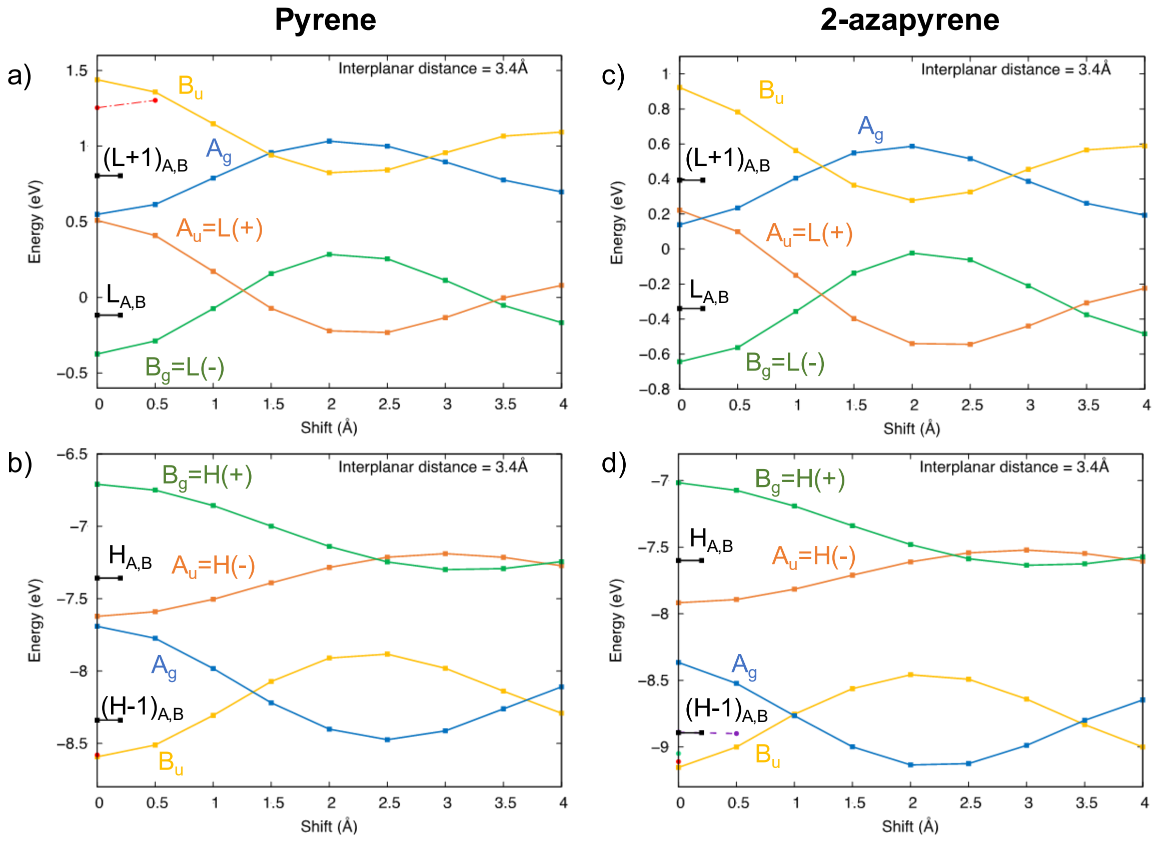

Figure S11. Energy profiles and symmetries of frontier molecular orbitals (MOs) of pyrene (a,b) and 2-azapyrene (c,d) dimers along the longitudinal shift. MOs of Bu (yellow), Ag (blue), Au (orange) and Bg (green) symmetries are derived from the linear combinations of monomers' frontier MOs (black lines, $(H-1)_{A,B}$; $H_{A,B}$; $L_{A,B}$; $(L+1)_{A,B}$) and are included in the diabatization procedure. The linear combinations of monomer orbitals (L(+/−) H(+/−) are also indicated for the lowest two occupied and highest two empty dimer orbitals. Dimer's MOs indicated by red, cyan and purple colors are of other nature and are excluded from the diabatization procedure. The interplanar distance considered here is 3.4Å. From TDA-ωB97X-D/def2-SVP calculations.

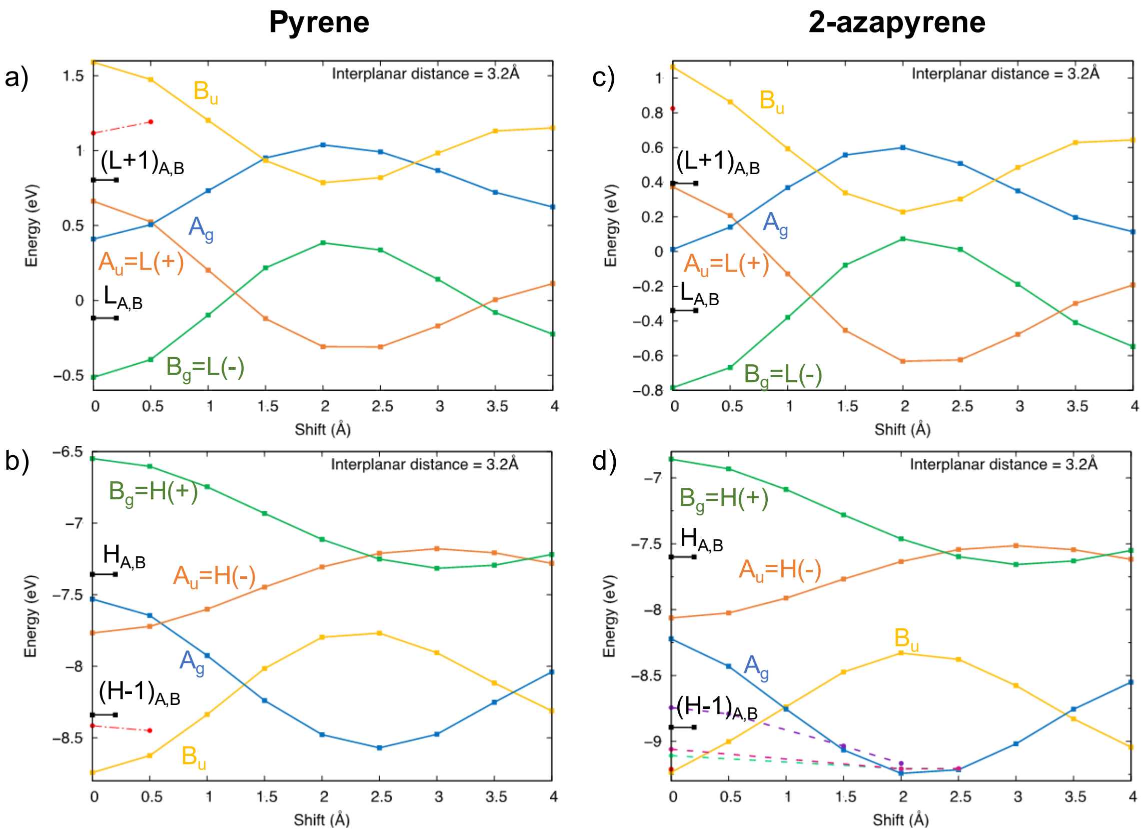

Figure S12. Energy profiles and symmetries of frontier molecular orbitals (MOs) of pyrene (a,b) and 2-azapyrene (c,d) dimers along the longitudinal shift. MOs of Bu (yellow), Ag (blue), Au (orange) and Bg (green) symmetries are derived from the linear combinations of monomers' frontier MOs (black lines, $(H-1)_{A,B}$; $H_{A,B}$; $L_{A,B}$; $(L+1)_{A,B}$) and are included in the diabatization procedure. The linear combinations of monomer orbitals (L(+/−) H(+/−) are also indicated for the lowest two occupied and highest two empty dimer orbitals. MOs indicated by red, cyan and purple colors are of other nature and are excluded from the diabatization procedure. The interplanar distance considered here is 3.2Å. From TDA-ωB97X-D/def2-SVP calculations.

Optimized cartesian coordinates for:

Pyrene dimer: ground state

C	-1.839747	-0.001702	-2.606916
C	-1.202683	1.205776	-2.338719
C	0.088505	1.229434	-1.796917
C	0.744548	-0.000838	-1.518893
C	0.088948	-1.231559	-1.796014
C	-1.202134	-1.208764	-2.338077
C	0.776644	2.461166	-1.502650
C	2.058413	-0.000392	-0.954877
C	2.710321	1.230941	-0.670644
C	2.022909	2.462536	-0.969237
C	3.990618	1.208906	-0.102535
C	4.621095	0.000493	0.180768
C	3.990984	-1.208365	-0.101491
C	2.710705	-1.231280	-0.669589
C	2.023692	-2.463351	-0.967176
C	0.777478	-2.462828	-1.500700
H	4.492071	2.154428	0.118827
H	-1.713011	2.150723	-2.539915
H	-1.711952	-2.154056	-2.538934
H	4.492738	-2.153539	0.120671
H	0.265580	-3.404583	-1.712538
H	2.531065	-3.405978	-0.746941
H	0.264437	3.402584	-1.715233
H	2.529987	3.405513	-0.749824
H	-2.851935	-0.002051	-3.015507
H	5.619311	0.000834	0.624224
C	-4.621495	0.000044	-0.179959
C	-3.990888	1.208770	0.101723
C	-2.710475	1.231502	0.669525
C	-2.058385	0.000510	0.954822
C	-2.710659	-1.230710	0.670916
C	-3.991201	-1.208474	0.103370
C	-2.023187	2.463451	0.966967
C	-0.744444	0.000733	1.518662
C	-0.088583	1.231335	1.795670
C	-0.776935	2.462717	1.500406
C	1.202592	1.208321	2.337521
C	1.840008	0.001167	2.606367
C	1.202555	-1.206202	2.338570
C	-0.088634	-1.229647	1.796777
C	-0.777076	-2.461265	1.502701
C	-2.023417	-2.462425	0.969476
H	1.712625	2.153541	2.538176
H	-4.492339	2.154058	-0.120661
H	-4.493007	-2.153925	-0.117497
H	1.712552	-2.151265	2.540050
H	-2.530719	-3.405322	0.750236
H	-0.265032	-3.402776	1.715270
H	-2.530409	3.406173	0.746786
H	-0.264879	3.404398	1.712192
H	-5.619888	-0.000097	-0.623005
H	2.852364	0.001332	3.014548

Pyrene excimer: eclipsed

C	3.530569	-0.001130	1.611481
C	2.844183	1.206548	1.603211
C	1.425039	1.233380	1.643768
C	0.711862	-0.000948	1.664471
C	1.424869	-1.235349	1.642694
C	2.844035	-1.208672	1.602302
C	0.689258	2.445714	1.637953
C	-0.711818	-0.000856	1.664517
C	-1.424833	1.233589	1.643968
C	-0.688878	2.445822	1.638126
C	-2.844001	1.206944	1.603415
C	-3.530512	-0.000607	1.611163
C	-2.844154	-1.208284	1.602050
C	-1.424986	-1.235166	1.642673
C	-0.689197	-2.447502	1.635706
C	0.688938	-2.447587	1.635673
H	-3.390735	2.152693	1.598474
H	3.391097	2.152195	1.598623
H	3.390788	-2.154407	1.596536
H	-3.391020	-2.153950	1.596027
H	1.235228	-3.393754	1.620877
H	-1.235591	-3.393605	1.620807
H	1.235670	3.391822	1.623917
H	-1.235132	3.392019	1.624150
H	4.622445	-0.001171	1.586228
H	-4.622379	-0.000511	1.585418
C	3.530580	0.000607	-1.611488
C	2.844207	1.208284	-1.601928
C	1.425063	1.235174	-1.642477
C	0.711873	0.000876	-1.664470
C	1.424865	-1.233555	-1.643983
C	2.844032	-1.206935	-1.603589
C	0.689295	2.447510	-1.635433
C	-0.711807	0.000986	-1.664516
C	-1.424810	1.235416	-1.642688
C	-0.688841	2.447634	-1.635615
C	-2.843980	1.208744	-1.602152
C	-3.530502	0.001209	-1.611162
C	-2.844155	-1.206484	-1.603313
C	-1.424987	-1.233339	-1.643951
C	-0.689213	-2.445690	-1.638212
C	0.688922	-2.445791	-1.638188
H	-3.390705	2.154492	-1.596228
H	3.391132	2.153919	-1.596344
H	3.390774	-2.152682	-1.598822
H	-3.391030	-2.152151	-1.598271
H	1.235201	-3.391978	-1.624348
H	-1.235618	-3.391801	-1.624264
H	1.235718	3.393596	-1.620440
H	-1.235084	3.393823	-1.620689
H	4.622456	0.000525	-1.586243
H	-4.622369	0.001285	-1.585417

Pyrene excimer: twisted

C	3.205745	-1.472265	1.473367
C	3.083174	-0.079697	1.478494
C	1.810236	0.531865	1.572626
C	0.648580	-0.294679	1.600694
C	0.780003	-1.715040	1.585879
C	2.084309	-2.280200	1.513096
C	1.647465	1.943686	1.597833
C	-0.646201	0.296339	1.600947
C	-0.777674	1.716636	1.585103
C	0.392382	2.513931	1.609911
C	-2.082078	2.281742	1.513195
C	-3.203540	1.473794	1.475197
C	-3.080942	0.081200	1.481013
C	-1.807888	-0.530252	1.574443
C	-1.645135	-1.942004	1.600941
C	-0.390013	-2.512283	1.612490
H	-2.184020	3.369410	1.500653
H	3.973939	0.551531	1.462711
H	2.186293	-3.367869	1.501134
H	-3.971653	-0.550117	1.466728
H	-0.283902	-3.599773	1.617540
H	-2.535739	-2.573585	1.608545
H	2.538151	2.575170	1.604101
H	0.286224	3.601422	1.614121
H	4.198517	-1.924679	1.417517
H	-4.196396	1.926122	1.420188
C	3.203380	1.472342	-1.475536
C	2.081966	2.280280	-1.515333
C	0.777642	1.715030	-1.587036
C	0.646198	0.294696	-1.601683
C	1.807912	-0.531816	-1.574023
C	3.080840	0.079759	-1.480380
C	-0.392420	2.512272	-1.612267
C	-0.648545	-0.296343	-1.600732
C	-1.810205	0.530215	-1.573020
C	-1.647501	1.941983	-1.599514
C	-3.083132	-0.081273	-1.478247
C	-3.205681	-1.473876	-1.472242
C	-2.084248	-2.281800	-1.511393
C	-0.779926	-1.716666	-1.584666
C	0.390107	-2.513928	-1.610489
C	1.645193	-1.943630	-1.599241
H	-3.973853	0.550019	-1.463137
H	2.183892	3.367955	-1.503654
H	3.971594	-0.551472	-1.464803
H	-2.186155	-3.369470	-1.498635
H	2.535834	-2.575169	-1.605958
H	0.283995	-3.601420	-1.614461
H	-0.286370	3.599769	-1.617088
H	-2.538094	2.573589	-1.605961
H	4.196185	1.924718	-1.419945
H	-4.198464	-1.926237	-1.416176

Pyrene excimer: perp

C	-3.554364	-0.150053	1.467459
C	-2.807313	-1.302320	1.593665
C	-1.386404	-1.243229	1.665538
C	-0.747116	0.034797	1.620261
C	-1.530634	1.221714	1.508461
C	-2.929920	1.105171	1.403092
C	-0.585856	-2.406551	1.751234
C	0.670509	0.121900	1.647318
C	1.455718	-1.068572	1.682194
C	0.789823	-2.328659	1.736336
C	2.858581	-0.959716	1.625773
C	3.482262	0.297322	1.599170
C	2.732826	1.454758	1.584427
C	1.309913	1.399903	1.599856
C	0.508927	2.564399	1.540919
C	-0.865001	2.482660	1.472239
H	3.462588	-1.870144	1.638303
H	-3.295401	-2.278992	1.632820
H	-3.532130	2.011520	1.304199
H	3.220785	2.431769	1.552780
H	-1.466392	3.392127	1.411051
H	1.000444	3.539219	1.510918
H	-1.077540	-3.380947	1.791154
H	1.391516	-3.238576	1.787000
H	-4.642918	-0.211896	1.401630
H	4.572754	0.354937	1.574522
C	-0.382800	-3.572221	-1.379726
C	-1.494085	-2.764018	-1.496919
C	-1.357242	-1.350592	-1.601722
C	-0.044896	-0.783692	-1.598872
C	1.098485	-1.630286	-1.494488
C	0.906500	-3.018254	-1.356792
C	-2.475764	-0.487688	-1.678924
C	0.120658	0.625795	-1.660970
C	-1.024361	1.476007	-1.688664
C	-2.320814	0.881351	-1.696870
C	-0.836036	2.871347	-1.671744
C	0.454146	3.423540	-1.687367
C	1.567829	2.610335	-1.677031
C	1.433065	1.192877	-1.655923
C	2.552029	0.329228	-1.600350
C	2.395209	-1.036051	-1.497202
H	-1.711407	3.525189	-1.679801
H	-2.497172	-3.196918	-1.503405
H	1.779833	-3.668292	-1.263860
H	2.571102	3.042848	-1.677856
H	3.270776	-1.686125	-1.440102
H	3.553091	0.765779	-1.600839
H	-3.476720	-0.924481	-1.685194
H	-3.196625	1.532138	-1.740686
H	-0.503808	-4.653942	-1.288586
H	0.573202	4.509295	-1.692194

2-azapyrene dimer: ground state

C	-0.705214	-2.464583	1.540659
C	-1.955819	-2.460308	1.014437
C	-2.627841	-1.219860	0.724629
C	-1.967166	-0.001269	1.011748
C	-0.653537	-0.001941	1.565550
C	-0.003991	-1.236568	1.832578
C	-2.627828	1.217996	0.727476
C	-3.905354	1.142400	0.156428
N	-4.522910	0.000063	-0.127052
C	-3.905369	-1.142937	0.153780
C	-0.004035	1.232046	1.835553
C	-0.705181	2.460766	1.546391
C	-1.955802	2.457767	1.020183
C	1.293582	1.207303	2.361843
C	1.932792	-0.003163	2.617522
C	1.293700	-1.213032	2.358716
H	1.808537	-2.156276	2.555909
H	-2.470947	3.396976	0.804148
H	-2.470973	-3.399005	0.796207
H	-4.444817	-2.066487	-0.088597
H	-4.444787	2.066515	-0.083818
H	2.952141	-0.003625	3.008273
H	1.808207	2.150085	2.561772
H	-0.197216	-3.409068	1.749288
H	-0.197111	3.404727	1.757212
C	0.705791	2.464689	-1.540697
C	1.956513	2.460176	-1.014754
C	2.628215	1.219584	-0.724830
C	1.967147	0.001141	-1.011705
C	0.653505	0.002066	-1.565485
C	0.004260	1.236834	-1.832545
C	2.627514	-1.218270	-0.727381
C	3.905063	-1.142981	-0.156356
N	4.522922	-0.000784	0.127037
C	3.905698	1.142361	-0.153915
C	0.003699	-1.231783	-1.835457
C	0.704615	-2.460638	-1.546354
C	1.955192	-2.457881	-1.020057
C	-1.293934	-1.206724	-2.361674
C	-1.932794	0.003892	-2.617534
C	-1.293402	1.213608	-2.358786
H	-1.807975	2.156975	-2.556078
H	2.470096	-3.397204	-0.803942
H	2.471796	3.398792	-0.796491
H	4.445414	2.065784	0.088345
H	4.444280	-2.067219	0.083899
H	-2.952125	0.004582	-3.008332
H	-1.808918	-2.149387	-2.561244
H	0.197815	3.409252	-1.749036
H	0.196393	-3.404514	-1.757184

2-azapyrene excimer: eclipsed

C	0.069353	2.447041	-0.619095
C	0.061304	2.443288	0.760586
C	0.059012	1.222954	1.480327
C	0.036699	0.000595	0.759586
C	0.045697	0.000322	-0.659884
C	0.077684	1.238093	-1.363996
C	0.059550	-1.221540	1.480785
C	0.117206	-1.140720	2.898907
N	0.118472	0.001071	3.574052
C	0.116808	1.142592	2.898506
C	0.078208	-1.237737	-1.363576
C	0.070358	-2.446385	-0.618210
C	0.062608	-2.442101	0.761485
C	0.158720	-1.210060	-2.781382
C	0.175451	-0.000097	-3.465182
C	0.158263	1.210029	-2.781830
H	0.180547	2.152958	-3.332578
H	0.072425	-3.384451	1.314380
H	0.070194	3.385821	1.313184
H	0.125243	2.067890	3.486404
H	0.125702	-2.065782	3.487160
H	0.250969	-0.000338	-4.553906
H	0.181610	-2.153119	-3.331884
H	0.085079	3.395406	-1.161350
H	0.086713	-3.394980	-1.160044
C	3.329988	-2.447245	0.618220
C	3.338891	-2.442998	-0.761475
C	3.341086	-1.222417	-1.480788
C	3.363228	-0.000270	-0.759599
C	3.354173	-0.000541	0.659872
C	3.321898	-1.238584	1.363562
C	3.340365	1.222085	-1.480332
C	3.282547	1.141715	-2.898519
N	3.281211	0.000228	-3.574039
C	3.283074	-1.141600	-2.898883
C	3.321897	1.237237	1.363981
C	3.329738	2.446184	0.619064
C	3.337596	2.442420	-0.760608
C	3.241689	1.209206	2.781813
C	3.224836	-0.000957	3.465195
C	3.241510	-1.210880	2.781403
H	3.219033	-2.153950	3.331913
H	3.328258	3.384941	-1.313219
H	3.330294	-3.385364	-1.314359
H	3.274467	-2.066650	-3.487151
H	3.273961	2.067027	-3.486399
H	3.149626	-0.001171	4.553940
H	3.219376	2.152125	3.332571
H	3.313684	-3.395832	1.160070
H	3.313702	3.394542	1.161321

2-azapyrene excimer: twisted

C	2.284340	1.029867	0.080707
C	1.400052	2.088535	0.114445
C	-0.000432	1.856897	0.154339
C	-0.477261	0.520772	0.111857
C	0.430326	-0.569936	0.088209
C	1.831848	-0.314440	0.096389
C	-1.877322	0.286990	0.144935
C	-2.721388	1.428929	0.272477
N	-2.280053	2.671214	0.332775
C	-0.963168	2.888224	0.292215
C	-0.070924	-1.903573	0.113828
C	-1.480121	-2.109254	0.100053
C	-2.357175	-1.042323	0.104364
C	0.853668	-2.970978	0.204481
C	2.230335	-2.718673	0.198989
C	2.719048	-1.425347	0.156486
H	3.795991	-1.241972	0.161548
H	-3.435643	-1.217846	0.110914
H	1.768517	3.116279	0.117460
H	-0.632539	3.931822	0.317863
H	-3.807889	1.283930	0.309346
H	2.927404	-3.558083	0.250673
H	0.480097	-3.996097	0.236882
H	3.360415	1.218830	0.067900
H	-1.858576	-3.133114	0.089453
C	-2.331760	1.088374	3.303801
C	-2.558289	-0.274106	3.300356
C	-1.478342	-1.185625	3.258688
C	-0.148778	-0.688333	3.289938
C	0.092274	0.709928	3.311048
C	-1.008528	1.614334	3.285640
C	0.931835	-1.607467	3.247803
C	0.612961	-2.982163	3.114531
N	-0.635957	-3.452261	3.074730
C	-1.639098	-2.596482	3.133314
C	1.433855	1.189118	3.300012
C	2.497642	0.250929	3.315995
C	2.260741	-1.108056	3.285243
C	1.646796	2.594808	3.236016
C	0.577112	3.470492	3.192904
C	-0.741526	3.000827	3.189751
H	-1.577611	3.701786	3.157710
H	3.093587	-1.814040	3.282426
H	-3.580146	-0.661057	3.296347
H	-2.652680	-3.013832	3.097350
H	1.420935	-3.720893	3.089166
H	0.761203	4.545809	3.138089
H	2.671198	2.974299	3.228599
H	-3.171309	1.785977	3.315369
H	3.524160	0.625072	3.326586

2-azapyrene excimer: perp

C	0.307330	2.127833	-0.399632
C	0.329557	1.989956	0.972404
C	0.208654	0.707104	1.591097
C	0.055515	-0.442361	0.762635
C	0.076365	-0.294745	-0.645475
C	0.212609	0.992225	-1.235150
C	-0.070056	-1.745320	1.334373
C	-0.037474	-1.866451	2.752773
C	0.268599	0.526258	2.986251
C	0.013563	-1.433926	-1.491211
C	-0.116464	-2.723617	-0.898805
C	-0.181567	-2.863989	0.473197
C	0.144442	-1.218906	-2.877585
C	0.288690	1.051094	-2.657573
H	0.384574	2.030745	-3.142233
H	-0.270901	-3.859641	0.912452
H	0.421831	2.871667	1.610063
H	0.396121	1.398066	3.632232
H	-0.137436	-2.858150	3.200083
H	0.122314	-2.075441	-3.560376
H	0.405373	3.115826	-0.854752
H	-0.187127	-3.601262	-1.544450
C	3.351739	-0.231316	-2.520841
C	3.159957	-1.591407	-2.377063
C	3.103953	-2.183672	-1.081924
C	3.245053	-1.349937	0.059335
C	3.395427	0.050399	-0.087134
C	3.444446	0.622565	-1.395349
C	3.181665	-1.938408	1.352188
C	2.966531	-3.346067	1.416216
N	2.811744	-4.115004	0.360467
C	2.857148	-3.552351	-0.857008
C	3.446838	0.875787	1.073343
C	3.397703	0.256752	2.360649
C	3.291069	-1.111564	2.493199
C	3.509165	2.272313	0.902162
C	3.577242	2.834704	-0.382839
C	3.545174	2.038513	-1.509760
H	3.589032	2.485474	-2.505676
H	3.248956	-1.566451	3.485255
H	3.074168	-2.229963	-3.258555
H	2.723041	-4.223705	-1.712417
H	2.921121	-3.830418	2.399640
H	3.643836	3.919758	-0.488545
H	3.538134	2.917024	1.783689
H	3.382247	0.208833	-3.519594
H	3.462942	0.889516	3.248286
N	0.256372	-0.005465	-3.439979
C	0.126907	-0.751929	3.550244
H	0.159968	-0.863643	4.636241

References:

1. Yamagata, H.; Pochas, C.M.; Spano, F.C. Designing J- and H-Aggregates through Wave Function Overlap Engineering: Applications to Poly(3-hexylthiophene). *J. Phys. Chem. B* **2012**, *116*, 14494–14503, doi:10.1021/jp309407r.
2. Canola, S.; Bagnara, G.; Dai, Y.; Ricci, G.; Calzolari, A.; Negri, F. Addressing the Frenkel and charge transfer character of exciton states with a model Hamiltonian based on dimer calculations: Application to large aggregates of perylene bisimide. *J. Chem. Phys.* **2021**, *154*, 124101, doi:10.1063/5.0045913.
3. Dai, Y.; Zubiria-Ulacia, M.; Casanova, D.; Negri, F. Impact of Charge-Resonance Excitations on CT-Mediated J-Type Aggregation in Singlet and Triplet Exciton States of Perylene Di-Imide Aggregates: A TDDFT Investigation. *Computation* **2022**, *10*, 18, doi:10.3390/computation10020018.
4. Norton, J.E.; Brédas, J.-L. Theoretical characterization of titanyl phthalocyanine as a p-type organic semiconductor: Short intermolecular π-π interactions yield large electronic couplings and hole transport bandwidths. *J. Chem. Phys.* **2008**, *128*, 034701, doi:10.1063/1.2806803.
5. Kim, D. A Theoretical Analysis of the Excited State of Oligoacene Aggregates: Local Excitation vs. Charge-Transfer Transition. *Bull. Korean Chem. Soc.* **2015**, *36*, 2284–2289, doi:10.1002/bkcs.10443.
6. Löwdin, P. On the Non-Orthogonality Problem Connected with the Use of Atomic Wave Functions in the Theory of Molecules and Crystals. *J. Chem. Phys.* **1950**, *18*, 365–375, doi:10.1063/1.1747632.
7. Casanova, D. Theoretical investigations of the perylene electronic structure: Monomer, dimers, and excimers. *Int. J. Quantum Chem.* **2015**, *115*, 442–452, doi:10.1002/qua.24869.
8. Liu, W.; Lunkenheimer, B.; Settels, V.; Engels, B.; Fink, R.F.; Köhn, A. A general ansatz for constructing quasi-diabatic states in electronically excited aggregated systems. *J. Chem. Phys.* **2015**, *143*, 084106, doi:10.1063/1.4929352.
9. Shirai, S.; Iwata, S.; Tani, T.; Inagaki, S. Ab Initio Studies of Aromatic Excimers Using Multiconfiguration Quasi-Degenerate Perturbation Theory. *J. Phys. Chem. A* **2011**, *115*, 7687–7699, doi:10.1021/jp201130k.
10. Nottoli, M.; Jurinovich, S.; Cupellini, L.; Gardiner, A.T.; Cogdell, R.; Mennucci, B. The role of charge-transfer states in the spectral tuning of antenna complexes of purple bacteria. *Photosynth. Res.* **2018**, *137*, 215–226, doi:10.1007/s11120-018-0492-1.
11. Becker, R.S.; Singh, I. Sen; Jackson, E.A. Comprehensive spectroscopic investigation of polynuclear aromatic hydrocarbons. I. Absorption spectra and state assignments for the tetracyclic hydrocarbons and their alkyl-substituted derivatives. *J. Chem. Phys.* **1963**, *38*, 2144–2171, doi:10.1063/1.1733946.
12. Kirchlechner, R.; Jutz, C. 2-Azapyrene. *Angew. Chem. Int. Ed. Engl.* **1968**, *7*, 376–377.
